# GISTIC2.0 facilitates sensitive and confident localization of the targets of focal somatic copy-number alteration in human cancers

**DOI:** 10.1186/gb-2011-12-4-r41

**Published:** 2011-04-28

**Authors:** Craig H Mermel, Steven E Schumacher, Barbara Hill, Matthew L Meyerson, Rameen Beroukhim, Gad Getz

**Affiliations:** 1 Cancer Program, The Broad Institute of MIT and Harvard, 7 Cambridge Center, Cambridge, MA 02142, USA; 2 Department of Medical Oncology, Dana Farber Cancer Institute, 44 Binney Street, Boston, MA 02115, USA; 3 Department of Cancer Biology, Dana Farber Cancer Institute, 44 Binney Street, Boston, MA 02115, USA; 4 The Center for Cancer Genome Discovery, Dana Farber Cancer Institute, 44 Binney Street, Boston, MA 02115, USA

## Abstract

We describe methods with enhanced power and specificity to identify genes targeted by somatic copy-number alterations (SCNAs) that drive cancer growth. By separating SCNA profiles into underlying arm-level and focal alterations, we improve the estimation of background rates for each category. We additionally describe a probabilistic method for defining the boundaries of selected-for SCNA regions with user-defined confidence. Here we detail this revised computational approach, GISTIC2.0, and validate its performance in real and simulated datasets.

## Background

Cancer forms through the stepwise acquisition of somatic genetic alterations, including point mutations, copy-number changes, and fusion events, that affect the function of critical genes regulating cellular growth and survival [1]. The identification of oncogenes and tumor suppressor genes being targeted by these alterations has greatly accelerated progress in both the understanding of cancer pathogenesis and the identification of novel therapeutic vulnerabilities [2]. Genes targeted by somatic copy-number alterations (SCNAs), in particular, play central roles in oncogenesis and cancer therapy [3]. Dramatic improvements in both array and sequencing platforms have enabled increasingly high-resolution characterization of the SCNAs present in thousands of cancer genomes [4-6].

However, the discovery of new cancer genes being targeted by SCNAs is complicated by two fundamental challenges. First, somatic alterations are acquired at random during each cell division, only some of which ('driver' alterations) promote cancer development [7]. Selectively neutral or weakly deleterious 'passenger' alterations may nonetheless become fixed whenever a subclone carrying such alterations acquires selectively beneficial mutations that promote clonal dominance [8]. Second, SCNAs may simultaneously affect up to thousands of genes, but the selective benefits of driver alterations are likely to be mediated by only one or a few of these genes. For these reasons, additional analysis and experimentation is required to distinguish the drivers from the passengers, and to identify the genes they are likely to target.

A common approach to identifying drivers is to study large collections of cancer samples, on the notion that regions containing driver events should be altered at higher frequencies than regions containing only passengers [4,6,7,9-14]. For example, we developed an algorithm, GISTIC (Genomic Identification of Significant Targets in Cancer) [15], that identifies likely driver SCNAs by evaluating the frequency and amplitude of observed events. GISTIC has been applied to multiple cancer types, including glioblastoma [10,15], lung adenocarcinoma [16], melanoma [17], colorectal carcinoma [18], hepatocellular carcinoma [19], ovarian carcinoma [20], medulloblastoma [21], and lung and esophageal squamous carcinoma [22], and has helped identify several new targets of amplifications (including *NKX2-1 *[16], *CDK8 *[18], *VEGFA *[19], *SOX2 *[22], and *MCL1 *and *BCL2L1 *[4]) and deletions (*EHMT1 *[21]). Several additional algorithms for identifying likely driver SCNAs have also been described [23-25] (reviewed in [26]).

Yet, several critical challenges have not yet been adequately addressed by any of the existing copy-number analysis tools. For example, we and others have shown that the abundance of SCNAs in human cancers varies according to their size, with chromosome-arm length SCNAs occurring much more frequently than SCNAs of slightly larger or smaller size [4,27]. Therefore, analysis methods need to model complex cancer genomes that contain a mixture of SCNA types occurring at distinct background rates. Existing copy-number methods have also used *ad hoc *heuristics to define the genomic regions likely to harbor true cancer gene targets. The inability of these methods to provide *a priori *statistical confidence has been a major limitation in interpreting copy-number analyses, an important problem as end-users typically use these results to prioritize candidate genes for time-consuming validation experiments.

Here we describe several methodological improvements to address these challenges, and validate the performance of the revised algorithms in both real and simulated datasets. We have incorporated these changes into a revised GISTIC pipeline, termed GISTIC 2.0.

## Results and Discussion

### Overview of copy-number analysis pipeline

**Figure 1:**
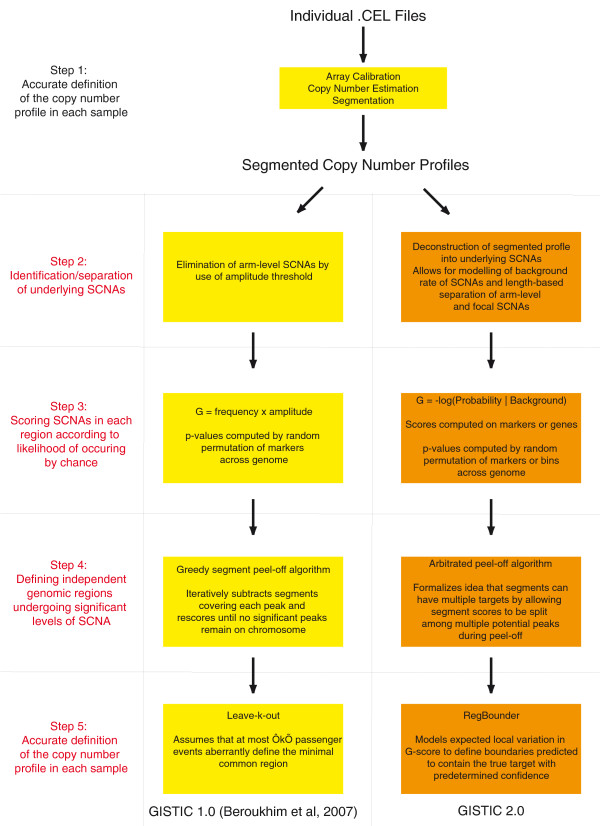
**Schematic overview of the copy-number analysis framework**. High-level overview of our cancer copy-number analysis framework, highlighting specific differences between the original GISTIC algorithm [15] and the GISTIC 2.0 pipeline described in this manuscript. The first step, accurate identification of the copy-number profile in each sample, is common to GISTIC and GISTIC2.0.

Cancer copy-number analyses can be divided into five discrete steps (Figure 1): 1) accurately defining the copy-number profile of each cancer sample; 2) identifying the SCNAs that most likely gave rise to these overall profiles and estimating their background rates of formation; 3) scoring the SCNAs in each region according to their likelihood of occurring by chance; 4) defining the independent genomic regions undergoing statistically significant levels of SCNA; and 5) identifying the likely gene target(s) of each significantly altered region. Figure 1 depicts a schematic overview of this process, highlighting the specific methodological improvements we will address in the present manuscript.

The first step, accurately defining the copy-number profile of each cancer sample, has been addressed by multiple previous studies [28-35] and is not discussed in detail here. We assume that segmented copy-number profiles have been obtained for all samples and all germline copy-number variations (CNVs) have been removed, yielding profiles of somatic events. The following sections describe improvements to steps 2 to 5. We evaluate these improvements on a test set of 178 glioblastoma multiforme (GBM) cancer DNAs hybridized to the Affymetrix Single Nucleotide Polymorphism (SNP) 6.0 array as part of The Cancer Genome Atlas (TCGA) project [10] (the 'TCGA GBM set'), and on simulated data. Full technical details for each step are described in the Supplementary Methods (Additional file 1).

### Deconstruction of segmented copy-number profiles into underlying SCNAs

Segmented copy number profiles represent the summed outcome of all the SCNAs that occurred during cancer development. Accurate modeling of the background rate of copy-number alteration requires analysis of the individual SCNAs. However, because SCNAs may overlap, it is impossible to directly infer the underlying events from the final segmented copy-number profile alone. Given certain assumptions about SCNA background rates, however, it is possible to estimate the likelihood of any given set of candidate SCNAs so as to select the most likely one.

We have developed an algorithm ('Ziggurat Deconstruction' (ZD)) that deconstructs each segmented copy-number profile into its most likely set of underlying SCNAs (see Supplementary Methods in Additional file 1 and Supplementary Figure S1 in Additional file 2). ZD is an iterative optimization algorithm that alternatively estimates a background model for SCNA formation and then utilizes this model to determine the most likely deconstruction of each copy-number profile. Its output is a catalog of the individual SCNAs in each cancer sample, each with an assigned length and amplitude, that sum to generate the original segmented copy profile. We assume that most of these SCNAs are passengers, so that their distribution reflects, to a first approximation, the operation of the 'background' mutation process (see Supplementary Figure S2 in Additional file 3).

### Length-based separation of focal and arm-level SCNAs

**Figure 2:**
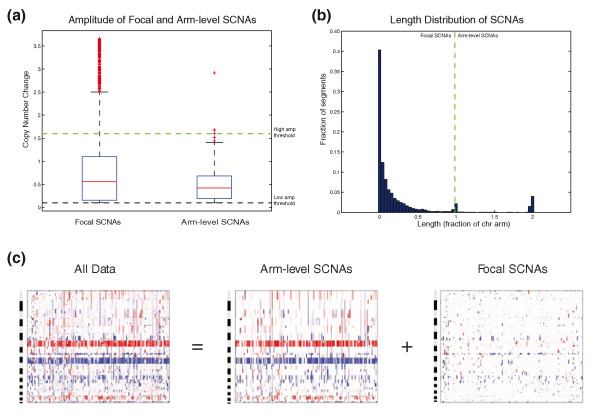
**Computational separation of arm-level and focal SCNAs**. **(a) **Boxplot showing the distribution of copy-number changes for amplified focal (length < 98% of a chromosome arm) and arm-level (length > 98% of a chromosome arm) SCNAs across 178 GBM profiles from TCGA. The black dotted line denotes a typical low-level amplitude threshold used to eliminate artifactual SCNAs, while the green dotted line denotes a typical high-level amplitude threshold used in previous version of GISTIC to eliminate arm-level SCNAs. **(b) **Histogram showing the frequency of observing SCNAs of a given length across 178 GBM samples. The high frequency of events occupying exactly one chromosome arm led us to distinguish between focal and arm-level SCNAs. **(c) **Heatmaps showing the total segmented copy-number profile of the TCGA GBM set (leftmost panel), and the results of computationally separating these samples into arm-level profiles (middle panel) and focal profiles (rightmost panel) by summing arm-level and focal SCNAs. In each heatmap, the chromosomes are arranged vertically from top to bottom and samples are arranged from left to right. Red and blue represent gain and loss, respectively.

A major advantage of the ZD method is its ability to separate arm-level and focal SCNAs explicitly by length. Prior studies have attempted to exclude arm-level SCNAs by setting high amplitude thresholds [10,16] because, in contrast to focal SCNAs, few arm-level SCNAs reach high amplitude (Figure 2a). However, this approach suffers from at least two undesirable consequences: first, low- to moderate-amplitude focal copy-number events are eliminated from the analysis, reducing sensitivity to identify positively selected regions; and second, the amplitude threshold is left as a free parameter, allowing for potential over-fitting of the analysis to a desired result.

We have previously shown that SCNA frequencies across cancers of diverse tissue origin are inversely proportional to SCNA lengths, with the striking exception of SCNAs exactly the length of a chromosome arm or whole chromosome (which are very frequent) [4]. This trend is preserved in the TCGA GBM samples (Figure 2b). This reproducible distribution provides a natural basis for classifying events as 'arm-level' and 'focal' based purely on length. Such length-based filtering of events allows for the computational reconstruction of 'arm-level' and 'focal' representations of the cancer genome (Figure 2c) and enables the inclusion of low- to moderate-amplitude focal copy-number events in the final analysis.

**Figure 3:**
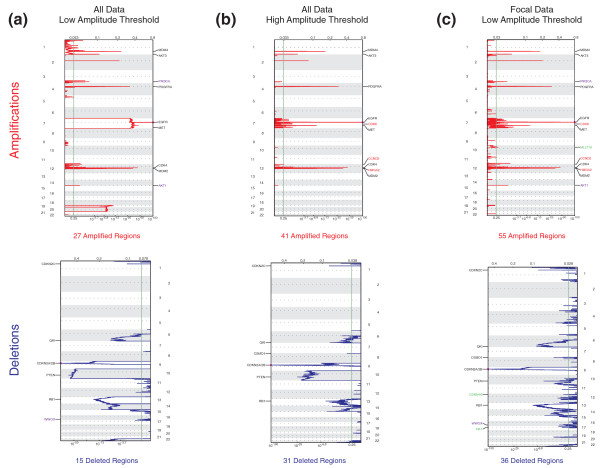
**Effects of amplitude-based or length-based filtering of arm-level events on GISTIC results**. **(a-c) **GISTIC amplification (top) and deletion (bottom) plots using all data and a low amplitude threshold (a), using all data and a high amplitude threshold (b), and using the focal data and a low amplitude threshold (c). The genome is oriented vertically from top to bottom, and GISTIC q-values at each locus are plotted from left to right on a log scale. The green line represents the significance threshold (q-value = 0.25). For each plot, known or interesting candidate genes are highlighted in black when identified by all three analyses, in red when identified by the high amplitude or focal length analyses, in purple when identified by the low amplitude or focal length analyses, and in green when identified only in the focal length analysis.

To determine the benefits of this approach, we ran the original 'GISTIC 1.0' algorithm on the TCGA GBM set using three different thresholding approaches (Figure 3; Supplementary Table S1 in Additional file 4): 1) a low amplitude threshold (log2 ratio of ± 0.1) that only eliminates low-level artifactual segments; 2) a high amplitude threshold (log2 ratio of 0.848 and -0.737 for amplifications/deletions) used previously [16] to eliminate arm-level events; and 3) the low amplitude threshold but also removing all SCNAs occupying more than 98% of a chromosome arm, leaving only the focal events.

Filtering out arm-level events through use of either amplitude or length thresholds greatly increased the sensitivity of GISTIC for detecting focal amplifications and deletions (Figure 3; Supplementary Table S1 in Additional file 4). While entire chromosomes were scored as significant using only a low amplitude threshold, including gain of chromosome 7 and loss of chromosome 10 (Figure 3a), a number of recurrent focal alterations were missed, including amplifications surrounding *CDK6*, *CCND2*, and *HMGA2*. These alterations were detected using either the high amplitude (Figure 3b) or the focal length filters (Figure 3c).

The benefits of length-based filtering result from the inclusion of low- to moderate-amplitude focal events. Amplification of *PIK3CA *and *AKT1 *and deletion of *WWOX *are detected using length-based filtering, but are not significant under the high amplitude filter (compare Figure 3b and 3c). Moreover, the length-based analysis identified significant SCNAs detected in neither of the amplitude-based analyses, including amplifications of *MLLT10 *and deletions of *CDKN1B *and *NF1*.

No known GBM target gene was detected in either of the amplitude-based analyses that was not also detected by the length-based analysis. These results suggest that length-based filtering of arm-level events greatly improves the sensitivity of GISTIC to identify relevant regions of focal SCNA.

### Probabilistic scoring of SCNAs

We set out to define a scoring framework for SCNAs that more accurately reflects the background rates of alteration. Ideally, we aim to score each region of the genome according to the probability with which the observed set of SCNAs would occur by chance alone. Scores using this framework have a clear interpretation: the higher the score assigned to a region, the less likely that the SCNAs in that region are observed entirely by chance, and the more likely that they underwent positive selection.

The probability of observing a single SCNA of given length and amplitude can be approximated by the frequency of occurrence of events of similar length and amplitude across the entire dataset (as in Supplementary Figure S2 in Additional file 3). However, since cancer genomes do contain drivers, this procedure is likely to overestimate the probability of observing SCNAs under the null model. Specifically, driver events tend to be shorter in length and of higher amplitude than passengers and therefore constitute the majority of events in their length/amplitude neighborhood (Supplementary Figure S3 in Additional file 5).

To avoid biasing our background model, we set out to fit the log-probability distribution of SCNAs to a functional form that would be insensitive to the presence of driver events in the data (Supplementary Methods in Additional file 1). We made use of a large collection of 3,131 cancer samples run on the Affymetrix 250K StyI SNP Array [4] plus several hundred additional samples run on the Affymetrix SNP6.0 Array (data not shown). At the level of resolution provided by these arrays, the probability of observing a focal SCNA at a given locus under the background model is roughly independent of length. As a result, the functional form for the log-probability distribution is similar to the original GISTIC G-score definition (G = Frequency × Amplitude), with the notable exception being that the new score is proportional to the amplitude in copy-number space rather than log-copy-number space.

Although this functional form was empirically derived from a large collection of samples run on two different array-based platforms, it does lead to increased sensitivity to differences in dynamic range across platforms as well as differential saturation characteristics of probes within the same array platform. To avoid this problem, we routinely cap the segmented copy-number data at a level representing the signal intensity above which most probes start to saturate (Supplementary Methods in Additional file 1). This ensures that we are using data that originate from the linear regime of the probes' response curves and therefore are more comparable across platforms.

As with GISTIC 1.0, we obtain *P*-values for each marker by comparing the score at each locus to a background score distribution generated by random permutation of the marker locations in each sample (Supplementary Methods in Additional file 1). This procedure controls for sample-specific variations in the rate of copy-number alteration. We correct the resulting *P*-values for multiple-hypothesis testing using the Benjamini-Hochberg false discovery rate method [36].

### Alternative gene-level scoring for tumor suppressors with non-overlapping deletions

Some genes are affected by non-overlapping deletions, either on different alleles in one sample or across multiple samples. For such genes, a marker-based score does not weight the presence of all deletions affecting that gene, despite the fact that these events are likely to have similarly deleterious effects on gene function. We have developed a modified scoring and permutation procedure, termed GeneGISTIC, that scores genes rather than markers (Supplementary Methods in Additional file 1). In each sample, we assign each gene the minimal copy number of any marker contained within that gene, and then sum across all samples to compute the gene score. Because genes covering more markers are more likely to achieve a more extreme value by chance, the permutation procedure is adjusted to account for gene size; the score for a gene covering *n *markers is compared against a size-specific null distribution generated by computing minima overall running windows of size *n *in each sample and then randomly permuting these minimal values across the genome.

To determine the effect of gene-based scoring of deletions, we compared the results of gene-based and marker-based scoring on the TCGA GBM set (holding all other parameters equal). As expected, GeneGISTIC ranks known tumor suppressor genes higher and is more sensitive for genes subject to non-overlapping deletions (Supplementary Table S2 in Additional file 6). For example, *RB1 *was ranked 5th out of 39 regions using gene-based scoring (q-value = 2.6e-10) but only 13th out of 38 using marker-based scoring (q-value = 0.0013), and *CDKN1B *was ranked 26th using gene-based scoring (q-value = 0.08) compared to 38th using marker-based scoring (q-value = 0.19). *NF1 *was focally deleted in 12 of the 178 GBM samples (6.7%), and these deletions were frequently non-overlapping (Supplementary Figure S4a in Additional file 7). As a result, *NF1 *was scored just over or just under the significance threshold using the marker-based score, depending on the parameters used. By contrast, *NF1 *was robustly identified using gene-based scoring across all parameter combinations (Supplementary Table S2 in Additional file 6 and data not shown).

However, because this scoring method does not score regions of the genome that are not in annotated genes, it could underweight or completely miss deletions occurring in non-genic regions. For example, in our GBM samples, gene-based scoring did not identify a region just outside of *PCHD9 *on chr13q21.3 that scored as highly significant (q-value = 4.4e-9) using the standard marker-based score (Supplementary Figure S4b in Additional file 7). While many non-genic deletions may in fact represent technical artifacts or rare germline events, some may be functionally relevant.

### Identification of independent significantly altered regions

Individual SCNAs, and indeed significantly amplified or deleted regions of the genome, may extend over more than one oncogene or tumor suppressor gene. Other significant regions may contain no oncogenes or tumor suppressor genes, but achieve apparent significance due to their proximity to a target gene. Thus, an additional step is required after genome-wide scoring to identify independently significant regions.

**Figure 4:**
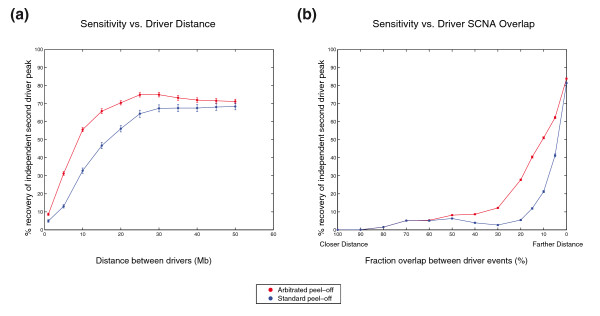
**Sensitivity of peel-off to detect secondary driver events**. The average fraction of secondary driver events recovered in independent (not containing the primary driver) peaks by GISTIC using the standard peel-off method (blue line) or arbitrated peel-off (red line) is shown for two simulated datasets. **(a) **The data are derived from 1,000 simulated chromosomes across 300 samples with a primary driver event present in 10% of samples and a secondary driver event a fixed distance away that is present in 5% of samples. **(b) **Data are derived from 10,000 simulated chromosomes across 300 samples with a primary driver event present in 10% of samples and a secondary driver event present in 5% of samples, where the fraction of the secondary driver events that overlapped with the primary driver event was varied between 100% (complete dependence; far left) and 0% (complete independence; far right). Error bars represent the mean ± standard error of the mean (some are too small to be visible).

GISTIC 1.0 solves this problem through the use of an iterative 'peel-off' algorithm, which greedily assigns all SCNAs to the maximal peak on each chromosome, removes them from the data, and rescores until no remaining region crosses the significance threshold. This approach reduces the power to identify secondary peaks that are close to previously identified significant regions (Figure 4a). However, since it is possible for individual SCNAs to affect multiple driver regions, a less greedy approach might identify additional peaks without significantly increasing the false discovery rate.

We have, therefore, modified the method to allow SCNAs to contribute to more than one peak ('arbitrated peel-off'). We first greedily assign the entirety of an SCNA's score to the most significant peak it covers. In subsequent steps, however, we allow scores of previously assigned segments to be redistributed before deciding whether a putative region is significant (Supplementary Methods in Additional file 1). Like the original algorithm, the process terminates when no region has an adjusted score that exceeds the significance threshold. A similar modification of GISTIC has recently been proposed [37].

Arbitrated peel-off is more sensitive than the original algorithm (Figure 4a; Supplementary Table S3 in Additional file 8). We generated 10,000 simulated datasets each consisting of 300 samples, with each chromosome containing a primary driver event in 10% of the samples and a secondary driver event in 5% of the samples. We analyzed the sensitivity of standard and arbitrated peel-off to detect the secondary peak as we varied the percentage of secondary driver events that overlapped the primary driver peak between 0% and 100% (Supplementary Methods in Additional file 1). At 0% overlap, the two methods were nearly equally sensitive at identifying the secondary peak. However, arbitrated peel-off was vastly more sensitive than standard peel-off as we increased the rate of overlap between primary and secondary peaks from 5 to 50% (Figure 4b), recovering an average of 2.4 times (range 1.2 to 3.8) more secondary peaks. Over 80% of the novel peaks identified by arbitrated peel-off corresponded to an actual simulated driver peak, demonstrating that the increased sensitivity is accompanied by high specificity.

The primary and secondary peaks tend to merge when the overlap is above 50%, obscuring any appreciable difference between the two methods (Supplementary Figure S5 in Additional file 9). Indeed, neither method was capable of independently identifying the secondary peak once the percent overlap rose above 80%. These simulations demonstrate both the superior sensitivity of arbitrated peel-off as well as the challenge of identifying neighboring drivers.

### Localizing target genes for each significantly altered region

**Figure 5:**
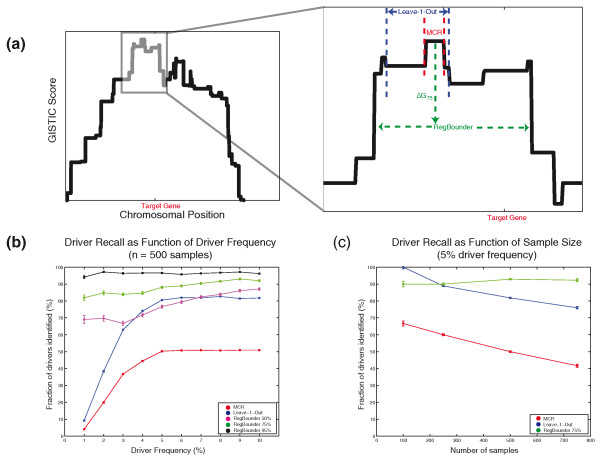
**Sensitivity of peak finding algorithms**. **(a) **Schematic diagram demonstrating various peak finding methods. The left panel shows the GISTIC score profile for a simulated chromosome containing a mix of driver events covering the denoted target gene and passenger events randomly scattered across the chromosome. The inset at right shows the region around the maximal G-score (gray box in left panel) in higher detail. The MCR (red dotted lines) is defined as the region of maximal segment overlap, or the region of highest G-score. The leave-k-out procedure (blue dotted lines, here shown for k = 1) is obtained by repeatedly computing the MCR after leaving out each sample in turn and taking as the left and right boundaries the minimal and maximal extent of the MCR. RegBounder works by attempting to find a region (dotted green line) over which the variation between boundary and maximal peak score is within the gth percentile of the local range distribution (Supplementary Methods in Additional file 1). Here, RegBounder produces a wider region than either the MCR or leave-k-out procedures, but is the only method whose boundary contains the true driver gene. **(b,c) **The average fraction of driver events contained within the peak region (conditional on having found a GISTIC peak within 10 Mb) is plotted as a function of driver-frequency (b) or sample size (c) for the MCR (red), leave-1-out (blue), and RegBounder algorithms (the latter at various confidence levels: 50%, magenta; 75%, green; 95%, black). In (b), data are derived from 10,000 simulated chromosomes across 500 samples in which the driver frequency varied from 1 to 10%. In (c), data are derived from 10,000 simulated chromosomes across a variable number of samples in which the driver frequency was fixed at 5%. Error-bars represent the mean ± standard error of the mean (some are too small to be visible).

The final step in the GISTIC pipeline is to determine the region that is most likely to contain the gene or genes being targeted for each independently significant region of SCNA (the 'peak region'). The standard approach is to focus on the minimal common region (MCR) of overlap (Figure 5a), the region that is altered in the greatest number of samples and therefore would be expected to be the most likely to contain the target genes. However, one or more passenger SCNAs adjacent to, but not overlapping, the target gene can result in an MCR that does not include the true target. This is a frequent occurrence, especially when the frequency of the driver event is low (< 5%; Figure 5b). An alternative method (utilized by the GISTIC 1.0) is to apply a heuristic 'leave-k-out' procedure to define the boundaries of each peak region (Figure 5a) [15]. This procedure assumes that up to k passenger SCNAs (typically, k = 1) may aberrantly define each boundary of the peak region. While the 'leave-k-out' procedure correctly identifies the target gene more often than the MCR (Figure 5b), it suffers from the potential for overfitting introduced by the free parameter 'k'. Moreover, the accuracy of 'leave-k-out' varies depending on the number of samples and the frequency of the event under question. For fixed k, the sensitivity of 'leave-k-out' increases for increasing driver frequency (Figure 5b) and decreases for increasing sample size (Figure 5c).

**Figure 6:**
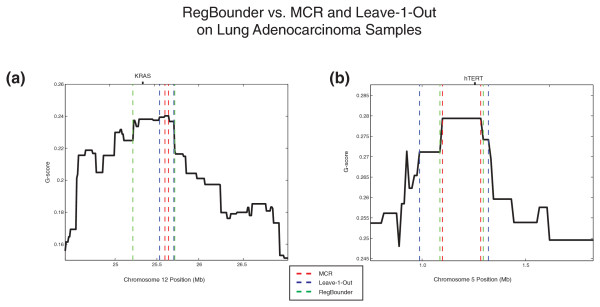
**Comparison of RegBounder to MCR and leave-1-out procedures applied to primary lung adenocarcinomas**. The advantages of RegBounder over previous peak-finding procedures are illustrated for two well-described oncogene peaks identified in GISTIC analysis of 371 lung adenocarcinoma samples characterized on the Affymetrix 250K StyI SNP array (as published in [16]). **(a) **A well-described amplification peak is identified on chromosome 12p12.1 with MCR (red dotted lines) near to but not containing the known lung cancer oncogene *KRAS*. Because there are more than two apparent passenger events in this region, the leave-1-out peak (blue dotted lines) also does not contain *KRAS*. However, RegBounder (green dotted lines) produces a wider peak that captures *KRAS*. **(b) **An amplification peak on chromosome 5p15.33 contains *hTERT*, the catalytic subunit of the human telomerase holoenzyme, within the MCR (red dotted lines). In this case, RegBounder (green dotted lines) produces a narrower peak region than the corresponding leave-1-out peak (blue dotted lines), demonstrating the ability of RegBounder to achieve a greater balance between peak region size and accuracy. In both (a) and (b), the y-axis depicts the amplification G-score and the x-axis denotes position along the corresponding chromosome.

We developed a novel approach (termed 'RegBounder') to define the peak region boundaries in such a way that target genes would be included at a pre-determined confidence level, regardless of the event frequency or number of samples being studied (Figure 5a; Supplementary Methods in Additional file 1). RegBounder models the expected random fluctuation in G-scores within any given window size and uses this distribution to define a confidence region likely to contain the true driver at least γ% of the time, where γ is a desired confidence level. Unlike the MCR and 'leave-k-out' procedures, which are highly dependent on one or a few segment boundaries to define each region, RegBounder is designed to be relatively robust to random errors (either due to technical artifacts or passenger segments) in boundary assignment. When applied to real data, RegBounder captures known driver genes more effectively than 'leave-1-out' (and MCR) in regions with increased local noise (Figure 6a) and yet is capable of producing narrower boundaries than 'leave-1-out' in regions with little noise (Figure 6b).

In simulated datasets, the performance of RegBounder was consistent across a wide range of driver SCNA frequencies (Figure 5b) and sample sizes (Figure 5c), and indeed controlled the probability of containing the driver. RegBounder captured the true driver gene in an average of 72%, 85%, and 95% of driver regions of varying frequency when run with a desired confidence level (γ) of 50, 75, and 95%, respectively. For no combination of sample-size, driver frequency, and γ did the average accuracy of RegBounder drop below γ.

**Figure 7:**
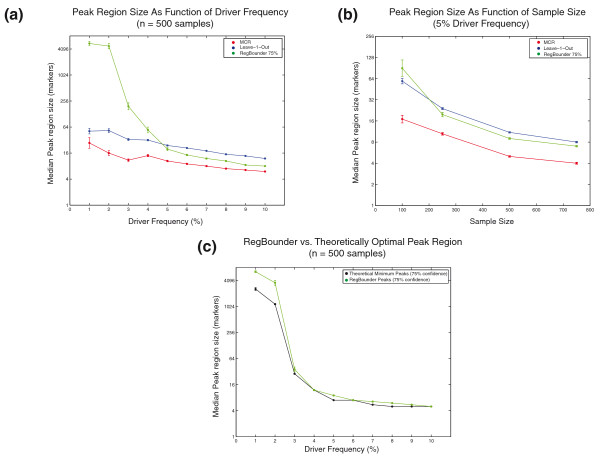
**Specificity of peak finding algorithms**. **(a,b) **The median size of the peak regions produced by the MCR (red), leave-1-out (blue), and RegBounder (green, 75% confidence) are shown as a function of driver frequency (a) and sample size (b). In (a), data are derived from 10,000 simulated chromosomes across 500 samples in which the driver frequency varied from 1 to 10%. In (b), data are derived from 10,000 simulated chromosomes across a variable number of samples in which the driver frequency was fixed at 5%. **(c) **Comparison of the peak region sizes obtained by RegBounder (green line) with the theoretically minimal peak region sizes (black line) that could be obtained by any peak finding algorithm with a similar confidence level (Supplementary Methods in Additional file 1). Error-bars represent the mean ± standard error of the mean (some are too small to be visible).

RegBounder also demonstrated a more optimal trade-off between peak region sensitivity (the likelihood of including the target gene) and specificity (the number of additional genes included) than the MCR or 'leave-k-out' approaches. The average size of the peak regions decreases with increasing driver frequency (Figure 7a) and sample size (Figure 7b) for all three approaches. However, RegBounder is more sensitive to these variables than the other methods, so that RegBounder peak regions (at 75% confidence) can range from an average of 90 times larger than the 'leave-k-out' peak regions (for datasets with few total driver events, in which the target gene locations are truly uncertain) to 37% smaller than the 'leave-k-out' procedure (for datasets with many total driver events). Thus, the increased confidence of RegBounder can even be achieved while producing narrower regions than the 'leave-k-out' procedure.

RegBounder is also more consistent across datasets than the MCR and 'leave-k-out' methods. We randomly split the TCGA GBM set into two groups and compared the peak regions produced by RegBounder and the MCR and 'leave-k-out' procedures on each. Considering only those peaks that were identified by GISTIC in both datasets, only 23% of the MCRs and 31% of the 'leave-k-out' peak regions overlap between the two datasets, reflecting the low confidence with which these regions are assigned. By contrast, a majority (53%) of the RegBounder peak regions (at 75% confidence) overlapped, as expected (0.75^2 ^= 56%). This increased overlap came with only a modestly increased median size of the RegBounder peak regions (370 kb) compared to the leave-k-out (163 kb) or MCR (115 kb) peak regions.

RegBounder regions are, on average, only 19% larger than the theoretically minimal peak region size for a wide range of driver frequencies (Figure 7c) and confidence levels (Supplementary Figure S6 in Additional file 10). These theoretically minimal peak region sizes were derived from the distribution of distances between the target gene and the MCR in our simulations (Supplementary Methods in Additional file 1). Our simulations reveal that RegBounder is capable of producing smaller peak regions than the 'leave-k-out' approach while simultaneously achieving greater target gene recall (compare Figures 5b and 7a; 'RegBounder 75%' versus 'leave-1-out', for driver frequencies > 5%). Thus, RegBounder is a robust algorithm for peak region boundary determination that demonstrates a more optimal trade-off between statistical confidence and peak resolution than previous heuristic approaches.

### Source code and module availability

The MATLAB source code for the GISTIC2.0 pipeline, along with a precompiled unix executable, will be available for download at [38]. In addition, the entire pipeline can be accessed through the GenePattern analysis portal at [39].

In addition to including all the methodological improvements described in this manuscript, the GISTIC2.0 source code has been designed to make efficient use of memory in storing segmented copy-number data (Supplementary Methods in Additional file 1). This improved memory efficiency should allow users with limited computational resources to run GISTIC2.0 on typical size datasets, and will be increasingly important for all users as the density of copy-number measuring platforms continues its rapid rise.

## Conclusions

We describe a number of analytical improvements to the standard copy-number analysis workflow that increase the sensitivity and specificity with which driver genes may be localized. We also demonstrate the utility of each of these changes using both simulated and real cancer copy-number datasets. While these changes have been specifically implemented in GISTIC 2.0, the challenges we describe apply broadly to the general task of identifying significantly aberrant regions of SCNA in cancer, and we anticipate that the approaches we have described can be adapted to other copy-number analysis workflows.

The procedure we outline enables data-driven estimation of the background rates of SCNA and how these rates vary with features of the SCNA, such as length or amplitude. The specific trends we have observed are likely to depend on the resolution and characteristics of the measuring platform used to generate our datasets (the Affymetrix 250K StyI and SNP6.0 arrays). As more cancer samples are characterized using higher-resolution array and sequencing platforms, new trends are likely to emerge. Further improvements would account for such trends, possibly taking into account additional features that may determine SCNA background rates, such as the presence of known fragile sites of the genome or the surrounding sequence context. Indeed, we and others have recently shown that somatic deletions frequently occur in genes with large genomic footprints [4,6], suggesting the existence of a contextual bias in the rate of somatic deletion that is presently unaccounted for in our background mutation model. Our probabilistic scoring framework allows such trends to be accounted for once the background model has been specified.

For the significant SCNAs, the background rate estimates also enable the delineation of regions likely to contain the target genes at predetermined confidence. RegBounder, the algorithm we devised to assign these boundaries, is more robust than either MCR- or 'leave-k-out'-based methods. RegBounder achieves this higher sensitivity by producing wider peak regions when the number of informative segments at a driver locus is small, but we find that RegBounder performs well compared to the theoretically optimal performance. However, RegBounder's underlying assumptions may not always be satisfied, including the assumption that each peak region contains a single dominant target gene and the expectation that copy-number breakpoints are independently distributed around the driver locus. To the extent that these assumptions are violated, RegBounder's performance may be worse than our simulations suggest.

While the arbitrated peel-off approach described in this manuscript reflects a more sensitive way of identifying independently targeted regions of amplification and deletion than our prior approach, it is still an imperfect attempt to decipher the complexity of cancer copy-number alterations. One major limitation stems from the fact that array-based measurements map SCNAs onto a linear reference genome. However, many SCNAs are preceded by rearrangement events that juxtapose genomic regions separated by great physical distance in the germline (even different chromosomes) [40,41]. This level of detailed structural information is impossible to infer from probe-level copy-number estimates but can be obtained by sequencing paired-end libraries [13]. Indeed, we anticipate that copy-number information derived from shotgun sequencing of cancer samples will become more common as sequencing costs continue to plummet [42]. Tools for estimating and segmenting copy-number values from sequencing coverage data already exist [5], and these segmented copy-number profiles can, with only slight modification, be run through the GISTIC 2.0 workflow. Fully exploiting the level of detailed information provided by these technologies will, however, require a significant extension of the background mutation model to include the probability of random genomic rearrangements, as well as the ability to perform significance analysis, segment peel-off, and peak finding across non-contiguous regions of the reference genome. The data provided by these sequencing efforts should lead to new insights into the cellular and molecular processes underlying SCNA generation in different cancer types, and will allow for the development of vastly more detailed and accurate models of the background mutation rate of such events during tumor development.

## Materials and methods

Full methods are available in the Supplementary Materials (Additional file 1) [43-46].

## Abbreviations

CNV: copy number variation; GBM: glioblastoma multiforme; GISTIC: Genomic Identification of Significant Targets in Cancer; MCR: minimal common region; SCNA: somatic copy number alteration; SNP: single nucleotide polymorphism; TCGA: The Cancer Genome Atlas; ZD: Ziggurat Deconstruction.

## Authors' contributions

RB and GG developed and coded the original GISTIC algorithm. CHM, SES, RB, and GG developed and coded the algorithmic modifications contained in GISTIC 2.0. CHM, MM, RB, and GG conceived and designed the present study. CHM, SES, and BH debugged and packaged the GISTIC 2.0 software release. CHM, MM, RB, and GG wrote the manuscript. All authors read and approved the final manuscript.

## Supplementary Material

Additional file 1 **Supplementary Methods**. Supplementary Methods contains the full description of the GISTIC2.0 method and details of the specific analyses presented in this manuscript. Click here for file

Additional file 2 **Supplementary Figure S1: Ziggurat Deconstruction**. **(a) **A hypothetical segmented chromosome (green line) is deconstructed with the simplified procedure used by Ziggurat Deconstruction (ZD) to initialize background SCNA rates. Dotted red and blue lines denote the length and amplitude of amplified and deleted SCNAs, respectively, while solid red and blue lines denote the result of merging the SCNA with the closest adjacent segment. **(b) **The same hypothetical segmented chromosome (green line) is deconstructed using the more flexible procedure of subsequent rounds of ZD. Here, the ZD is performed with respect to up to two basal levels (dotted magenta lines) that are fit to the data, allowing for amplified and deleted SCNAs to be superimposed. Click here for file

Additional file 3 **Supplementary Figure S2: distribution of SCNA length and amplitudes**. Two-dimensional histogram showing the frequency (z-axis) of copy number events as a function of length (x-axis) and amplitude (y-axis). Frequency is plotted on a log-scale to facilitate visualization of very low frequency copy number events. Click here for file

Additional file 4 **Supplementary Table S1: comparison of amplitude and length-based filtering of SCNAs**. Supplementary Table 1 compares the GISTIC results obtained using low and high amplitude thresholds with those obtained using a focal length threshold on 178 GBM samples. Click here for file

Additional file 5 **Supplementary Figure S3: distribution of driver length and amplitudes**. Driver SCNAs are typically of shorter length and higher amplitude than random passenger SCNAs. **(a,b) **Here we show the cumulative frequency distribution of SCNA amplitudes (a) and lengths (b) for SCNAs covering significantly amplified regions identified by GISTIC ('Driver SCNAs', red line) or by a similar number of randomly chosen non-driver regions ('Random SCNAs', blue line). Click here for file

Additional file 6 **Supplementary Table S2: comparison of GeneGISTIC and standard GISTIC deletions analysis**. Supplementary Table 2 compares the GISTIC results obtained using the standard GISTIC deletions analysis with those obtained using GeneGISTIC on 178 GBM sanples. Click here for file

Additional file 7 **Supplementary Figure S4: GeneGISTIC versus standard GISTIC**. **(a) **GeneGISTIC helps identify genes subject to non-overlapping deletion, such as *NF1*. The left panel shows the 12 samples with focal deletions affecting *NF1*, many of which do not overlap. As a result, the standard GISTIC marker score (blue line, right panel) has multiple local maxima over *NF1*. By contrast, the GeneGISTIC score counts all of these deletions as contributing to the *NF1 *score, resulting in a score for *NF1 *(red line, right panel) that is significantly greater than that assigned to any of the individual markers covering NF1. **(b) **GeneGISTIC does not score deletions occurring outside of genes. The left panel shows a region of focal deletion occurring just outside the *PCHD9 *gene on chromosome 13. These deletions result in a peak in the markers deletion score (blue line, right panel) that is not detected by GeneGISTIC. Click here for file

Additional file 8 **Supplementary Table S3: new peaks detected by arbitrated peel-off**. Supplementary Table 3 compares the GISTIC results obtained using the standard peel-off algorithm with those obtained using arbitrated peel-off on 178 GBM samples. Click here for file

Additional file 9 **Supplementary Figure S5: total recovery of secondary driver peaks**. This figure shows the results from 10,000 simulations of 300 samples in which a primary driver event is present in 10% of samples and a secondary driver event is present in 5% of samples. In these simulations, we vary the fraction of overlap between driver events from 100% (total dependence) to 0% (total independence). Here we present to the total recovery of the secondary driver peak in GISTIC runs using arbitrated peel-off (left panel) or the standard peel-off (right panel). The red (left panel) or blue (right panel) lines show the fraction of secondary driver peaks identified in independent GISTIC peaks (that is, not containing the primary driver event), as is shown in Figure 4b. The black lines show the fraction of secondary driver peaks identified in dependent peaks (that is, a peak containing both the primary and secondary driver events), and the green lines show the total recall of secondary driver peaks (in any peak). Error-bars representing the mean ± standard error of the mean are drawn, but may be smaller than the point used to represent the mean and hence not be visible. Click here for file

Additional file 10 **Supplementary Figure S6: comparison of RegBounder to theoretically optimal peaks**. Comparison between the peak region sizes obtained by RegBounder (green line) with the theoretically minimal peak region sizes (black line) that could be obtained by a similarly confident peak finding algorithm (Supplementary Methods in Additional file 1) at 50% (left) and 95% (right) confidence. Error-bars representing the median ± standard error of the mean are drawn, but may be smaller than the points used to represent the median and hence not be visible. Click here for file

## References

[B1] Hanahan D Weinberg RA The hallmarks of cancer. Cell 2000 100 57 70 10.1016/S0092-8674(00)81683-9 10647931

[B2] Stratton MR Campbell PJ Futreal PA The cancer genome. Nature 2009 458 719 724 10.1038/nature07943 19360079 PMC2821689

[B3] Santarius T Shipley J Brewer D Stratton MR Cooper CS A census of amplified and overexpressed human cancer genes. Nat Rev Cancer 2010 10 59 64 10.1038/nrc2771 20029424

[B4] Beroukhim R Mermel C Porter D Wei G Raychaudhuri S Donovan J Barretina J Boehm J Dobson J Urashima M The landscape of somatic copy-number alteration across human cancers. Nature 2010 463 899 905 10.1038/nature08822 20164920 PMC2826709

[B5] Chiang D Getz G Jaffe D O'Kelly M Zhao X High-resolution mapping of copy-number alterations with massively parallel sequencing. Nat Methods 2009 6 99 103 10.1038/nmeth.1276 19043412 PMC2630795

[B6] Bignell GR Greenman CD Davies H Butler AP Edkins S Andrews JM Buck G Chen L Beare D Latimer C Widaa S Hinton J Fahey C Fu B Swamy S Dalgliesh GL Teh BT Deloukas P Yang F Campbell PJ Futreal PA Stratton MR Signatures of mutation and selection in the cancer genome. Nature 2010 463 893 898 10.1038/nature08768 20164919 PMC3145113

[B7] Greenman C Stephens P Smith R Dalgliesh GL Hunter C Bignell G Davies H Teague J Butler A Stevens C Edkins S O'Meara S Vastrik I Schmidt EE Avis T Barthorpe S Bhamra G Buck G Choudhury B Clements J Cole J Dicks E Forbes S Gray K Halliday K Harrison R Hills K Hinton J Jenkinson A Jones D Patterns of somatic mutation in human cancer genomes. Nature 2007 446 153 158 10.1038/nature05610 17344846 PMC2712719

[B8] Merlo LM Pepper JW Reid BJ Maley CC Cancer as an evolutionary and ecological process. Nat Rev Cancer 2006 6 924 935 10.1038/nrc2013 17109012

[B9] Network CGAR Comprehensive genomic characterization defines human glioblastoma genes and core pathways. Nature 2008 455 1061 1068 10.1038/nature07385 18772890 PMC2671642

[B10] McLendon R Friedman A Bigner D Van Meir EG Brat DJ Mastrogianakis GM Olson JJ Mikkelsen T Lehman N Aldape K Yung WK Bogler O Weinstein JN VandenBerg S Berger M Prados M Muzny D Morgan M Scherer S Sabo A Nazareth L Lewis L Hall O Zhu Y Ren Y Alvi O Yao J Hawes A Jhangiani S Fowler G Comprehensive genomic characterization defines human glioblastoma genes and core pathways. Nature 2008 455 1061 1068 10.1038/nature07385 18772890 PMC2671642

[B11] Pleasance E Cheetham R Stephens P McBride D Humphray S Greenman C Varela I Lin M Ordóñez G Bignell G A comprehensive catalogue of somatic mutations from a human cancer genome. Nature 2009 463 191 196 20016485 10.1038/nature08658 PMC3145108

[B12] Pleasance ED Stephens PJ O'Meara S McBride DJ Meynert A Jones D Lin ML Beare D Lau KW Greenman C Varela I Nik-Zainal S Davies HR Ordonez GR Mudie LJ Latimer C Edkins S Stebbings L Chen L Jia M Leroy C Marshall J Menzies A Butler A Teague JW Mangion J Sun YA McLaughlin SF Peckham HE Tsung EF A small-cell lung cancer genome with complex signatures of tobacco exposure. Nature 2010 463 184 190 10.1038/nature08629 20016488 PMC2880489

[B13] Stephens PJ McBride DJ Lin ML Varela I Pleasance ED Simpson JT Stebbings LA Leroy C Edkins S Mudie LJ Greenman CD Jia M Latimer C Teague JW Lau KW Burton J Quail MA Swerdlow H Churcher C Natrajan R Sieuwerts AM Martens JW Silver DP Langerod A Russnes HE Foekens JA Reis-Filho JS van 't Veer L Richardson AL Borresen-Dale AL Complex landscapes of somatic rearrangement in human breast cancer genomes. Nature 2009 462 1005 1010 10.1038/nature08645 20033038 PMC3398135

[B14] Sjoblom T Jones S Wood LD Parsons DW Lin J Barber TD Mandelker D Leary RJ Ptak J Silliman N Szabo S Buckhaults P Farrell C Meeh P Markowitz SD Willis J Dawson D Willson JK Gazdar AF Hartigan J Wu L Liu C Parmigiani G Park BH Bachman KE Papadopoulos N Vogelstein B Kinzler KW Velculescu VE The consensus coding sequences of human breast and colorectal cancers. Science 2006 314 268 274 10.1126/science.1133427 16959974

[B15] Beroukhim R Getz G Nghiemphu L Barretina J Hsueh T Linhart D Vivanco I Lee JC Huang JH Alexander S Du J Kau T Thomas RK Shah K Soto H Perner S Prensner J Debiasi RM Demichelis F Hatton C Rubin MA Garraway LA Nelson SF Liau L Mischel PS Cloughesy TF Meyerson M Golub TA Lander ES Mellinghoff IK Assessing the significance of chromosomal aberrations in cancer: methodology and application to glioma. Proc Natl Acad Sci USA 2007 104 20007 20012 10.1073/pnas.0710052104 18077431 PMC2148413

[B16] Weir BA Woo MS Getz G Perner S Ding L Beroukhim R Lin WM Province MA Kraja A Johnson LA Shah K Sato M Thomas RK Barletta JA Borecki IB Broderick S Chang AC Chiang DY Chirieac LR Cho J Fujii Y Gazdar AF Giordano T Greulich H Hanna M Johnson BE Kris MG Lash A Lin L Lindeman N Characterizing the cancer genome in lung adenocarcinoma. Nature 2007 450 893 898 10.1038/nature06358 17982442 PMC2538683

[B17] Lin WM Baker AC Beroukhim R Winckler W Feng W Marmion JM Laine E Greulich H Tseng H Gates C Hodi FS Dranoff G Sellers WR Thomas RK Meyerson M Golub TR Dummer R Herlyn M Getz G Garraway LA Modeling genomic diversity and tumor dependency in malignant melanoma. Cancer Res 2008 68 664 673 10.1158/0008-5472.CAN-07-2615 18245465 PMC10493008

[B18] Firestein R Bass AJ Kim SY Dunn IF Silver SJ Guney I Freed E Ligon AH Vena N Ogino S Chheda MG Tamayo P Finn S Shrestha Y Boehm JS Jain S Bojarski E Mermel C Barretina J Chan JA Baselga J Tabernero J Root DE Fuchs CS Loda M Shivdasani RA Meyerson M Hahn WC CDK8 is a colorectal cancer oncogene that regulates beta-catenin activity. Nature 2008 455 547 551 10.1038/nature07179 18794900 PMC2587138

[B19] Chiang DY Villanueva A Hoshida Y Peix J Newell P Minguez B LeBlanc AC Donovan DJ Thung SN Sole M Tovar V Alsinet C Ramos AH Barretina J Roayaie S Schwartz M Waxman S Bruix J Mazzaferro V Ligon AH Najfeld V Friedman SL Sellers WR Meyerson M Llovet JM Focal gains of VEGFA and molecular classification of hepatocellular carcinoma. Cancer Res 2008 68 6779 6788 10.1158/0008-5472.CAN-08-0742 18701503 PMC2587454

[B20] Etemadmoghadam D deFazio A Beroukhim R Mermel C George J Getz G Tothill R Okamoto A Raeder MB Harnett P Lade S Akslen LA Tinker AV Locandro B Alsop K Chiew YE Traficante N Fereday S Johnson D Fox S Sellers W Urashima M Salvesen HB Meyerson M Bowtell D Bowtell D Chenevix-Trench G Green A Webb P deFazio A Integrated genome-wide DNA copy number and expression analysis identifies distinct mechanisms of primary chemoresistance in ovarian carcinomas. Clin Cancer Res 2009 15 1417 1427 10.1158/1078-0432.CCR-08-1564 19193619 PMC2670486

[B21] Northcott PA Nakahara Y Wu X Feuk L Ellison DW Croul S Mack S Kongkham PN Peacock J Dubuc A Ra Y-S Zilberberg K McLeod J Scherer SW Sunil Rao J Eberhart CG Grajkowska W Gillespie Y Lach B Grundy R Pollack IF Hamilton RL Van Meter T Carlotti CG Boop F Bigner D Gilbertson RJ Rutka JT Taylor MD Multiple recurrent genetic events converge on control of histone lysine methylation in medulloblastoma. Nat Genet 2009 41 465 472 10.1038/ng.336 19270706 PMC4454371

[B22] Bass AJ Watanabe H Mermel CH Yu S Perner S Verhaak RG Kim SY Wardwell L Tamayo P Gat-Viks I Ramos AH Woo MS Weir BA Getz G Beroukhim R O'Kelly M Dutt A Rozenblatt-Rosen O Dziunycz P Komisarof J Chirieac LR Lafargue CJ Scheble V Wilbertz T Ma C Rao S Nakagawa H Stairs DB Lin L Giordano TJ SOX2 is an amplified lineage-survival oncogene in lung and esophageal squamous cell carcinomas. Nat Genet 2009 41 1238 1242 10.1038/ng.465 19801978 PMC2783775

[B23] Diskin SJ Eck T Greshock J Mosse YP Naylor T Stoeckert CJ Weber BL Maris JM Grant GR STAC: A method for testing the significance of DNA copy number aberrations across multiple array-CGH experiments. Genome Res 2006 16 1149 1158 10.1101/gr.5076506 16899652 PMC1557772

[B24] Guttman M Mies C Dudycz-Sulicz K Diskin SJ Baldwin DA Stoeckert CJ Grant GR Assessing the significance of conserved genomic aberrations using high resolution genomic microarrays. PLoS Genet 2007 3 e143 10.1371/journal.pgen.0030143 17722985 PMC1950957

[B25] Taylor BS Barretina J Socci ND Decarolis P Ladanyi M Meyerson M Singer S Sander C Gibson G Functional copy-number alterations in cancer. PLoS ONE 2008 3 e3179 10.1371/journal.pone.0003179 18784837 PMC2527508

[B26] Shah SP Computational methods for identification of recurrent copy number alteration patterns by array CGH. Cytogenet Genome Res 2008 123 343 351 10.1159/000184726 19287173

[B27] Leach NT Rehder C Jensen K Holt S Jackson-Cook C Human chromosomes with shorter telomeres and large heterochromatin regions have a higher frequency of acquired somatic cell aneuploidy. Mech Ageing Dev 2004 125 563 573 10.1016/j.mad.2004.06.006 15336914

[B28] Li C Hung Wong W Model-based analysis of oligonucleotide arrays: model validation, design issues and standard error application. Genome Biol 2001 2 RESEARCH0032 11532216 10.1186/gb-2001-2-8-research0032 PMC55329

[B29] Li C Wong WH Model-based analysis of oligonucleotide arrays: expression index computation and outlier detection. Proc Natl Acad Sci USA 2001 98 31 36 10.1073/pnas.011404098 11134512 PMC14539

[B30] Bolstad BM Collin F Simpson KM Irizarry RA Speed TP Experimental design and low-level analysis of microarray data. Int Rev Neurobiol 2004 60 25 58 15474586 10.1016/S0074-7742(04)60002-X

[B31] Baross A Delaney AD Li HI Nayar T Flibotte S Qian H Chan SY Asano J Ally A Cao M Birch P Brown-John M Fernandes N Go A Kennedy G Langlois S Eydoux P Friedman JM Marra MA Assessment of algorithms for high throughput detection of genomic copy number variation in oligonucleotide microarray data. BMC Bioinformatics 2007 8 368 10.1186/1471-2105-8-368 17910767 PMC2148068

[B32] Hupé P Stransky N Thiery J-P Radvanyi F Barillot E Analysis of array CGH data: from signal ratio to gain and loss of DNA regions. Bioinformatics 2004 20 3413 3422 10.1093/bioinformatics/bth418 15381628

[B33] Olshen AB Venkatraman ES Lucito R Wigler M Circular binary segmentation for the analysis of array-based DNA copy number data. Biostatistics 2004 5 557 572 10.1093/biostatistics/kxh008 15475419

[B34] Venkatraman ES Olshen AB A faster circular binary segmentation algorithm for the analysis of array CGH data. Bioinformatics 2007 23 657 663 10.1093/bioinformatics/btl646 17234643

[B35] Nilsson B Johansson M Al-Shahrour F Carpenter AE Ebert BL Ultrasome: efficient aberration caller for copy number studies of ultra-high resolution. Bioinformatics 2009 25 1078 1079 10.1093/bioinformatics/btp091 19228802

[B36] Benjamini Y Hochberg Y Controlling the false discovery rate: a practical and powerful approach to multiple testing. J R Stat Soc B (Methodological) 1995 57 289 300

[B37] Sanchez-Garcia F Akavia UD Mozes E Pe'er D JISTIC: identification of significant targets in cancer. BMC Bioinformatics 2010 11 189 10.1186/1471-2105-11-189 20398270 PMC2873534

[B38] GISTIC 2 Manuscript and Software Download Page http://www.broadinstitute.org/cancer/pub/GISTIC2

[B39] GenePattern http://www.broadinstitute.org/cancer/software/genepattern/

[B40] Stephens PJ Greenman CD Fu B Yang F Bignell GR Mudie LJ Pleasance ED Lau KW Beare D Stebbings LA McLaren S Lin ML McBride DJ Varela I Nik-Zainal S Leroy C Jia M Menzies A Butler AP Teague JW Quail MA Burton J Swerdlow H Carter NP Morsberger LA Iacobuzio-Donahue C Follows GA Green AR Flanagan AM Stratton MR Massive genomic rearrangement acquired in a single catastrophic event during cancer development. Cell 2011 144 27 40 10.1016/j.cell.2010.11.055 21215367 PMC3065307

[B41] Dahlback HS Brandal P Meling TR Gorunova L Scheie D Heim S Genomic aberrations in 80 cases of primary glioblastoma multiforme: Pathogenetic heterogeneity and putative cytogenetic pathways. Genes Chromosomes Cancer 2009 48 908 924 10.1002/gcc.20690 19603525

[B42] Metzker M Sequencing technologies - the next generation. Nat Rev Genet 2009 11 31 46 19997069 10.1038/nrg2626

[B43] The Cancer Genome Atlas Data Portal, GBM Publication http://tcga-data.nci.nih.gov/docs/publications/gbm_2008/

[B44] McCarroll SA Kuruvilla FG Korn JM Cawley S Nemesh J Wysoker A Shapero MH de Bakker PI Maller JB Kirby A Elliott AL Parkin M Hubbell E Webster T Mei R Veitch J Collins PJ Handsaker R Lincoln S Nizzari M Blume J Jones KW Rava R Daly MJ Gabriel SB Altshuler D Integrated detection and population-genetic analysis of SNPs and copy number variation. Nat Genet 2008 40 1166 1174 10.1038/ng.238 18776908

[B45] Schwarz G Estimating the dimension of a model. Ann Statist 1978 6 461 464 10.1214/aos/1176344136

[B46] Holland AJ Cleveland DW Boveri revisited: chromosomal instability, aneuploidy and tumorigenesis. Nat Rev Mol Cell Biol 2009 10 478 487 19546858 10.1038/nrm2718 PMC3154738

