# Supplementary material for: GISTIC2.0 facilitates sensitive and confident localization of the targets of focal somatic copy-number alteration in human cancers
Source: Genome Biol. 2011 Apr 28;12(4):R41. doi: 10.1186/gb-2011-12-4-r41 (PMC3218867; doi:10.1186/gb-2011-12-4-r41)
Supplement: Additional file 1 — Supplementary Methods. Supplementary Methods contains the full description of the GISTIC2.0 method and details of the specific analyses presented in this manuscript. [file gb-2011-12-4-r41-S1.DOC]

**Supplementary Methods**

1. **Generation and post-processing of segmented data from Affymetrix SNP6.0 arrays**

SNP6.0 data were generated at the Broad Institute as part of The Cancer Genome Atlas (TCGA) Pilot Project, and the raw CEL files were normalized to copy number estimates using a GenePattern pipeline (detailed in [10] and Monte et al, manuscript in preparation). Normalized copy number estimates (log_2_ ratios) were segmented using the Circular Binary Segmentation (CBS) algorithm, followed by median centering the segment values in each sample around 0. These data were obtained from the Level 3 segmented copy number data files that are available for public download from the TCGA Data Portal website at [43].

We additionally removed markers residing within previously annotated regions of germline copy number variation [44] and merged segments with fewer than 10 markers with the closest adjacent segment. We felt that most of these very small segments are likely to have been falsely separated.

1. **Deconstruction of segmented copy profiles into ‘underlying’ SCNAs**

As described in the main text, we apply an algorithm termed “Ziggurat Deconstruction” (ZD) to the segmented copy data in an attempt to reconstruct the most likely sequence of underlying copy number events needed to explain each segmented copy profile. Ziggurat Deconstruction bears a certain analogy to segmentation algorithms, which attempt to maximize the likelihood of the probe-level data on each chromosome given a proposed segmentation model (plus a penalty for increasing model complexity, see Box 1). Ziggurat Deconstruction attempts to maximize the likelihood of observing the segmented copy data on each chromosome given a proposed SCNA history (plus a penalty for model complexity, see Box 2).

**BOX1: Segmentation Algorithm**

**Goal:**


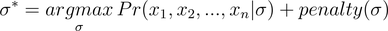


**Given:**

Probe level data x_1_,x_2_, … x_n_ and a proposed segmentation σ.

**BOX2: Ziggurat Deconstruction**

**Goal:**

**
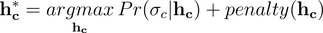
**

**Given:**

A chromosomal segmentation profile σ_c_ and proposed SCNA history **h**_c_.

ZD performs this likelihood maximization by iterating between two complementary procedures:

**Deconstruction:** Converts segmentation profiles into the most likely history of underlying SCNAs, using an estimate for the background rate of SCNAs as a function of length and amplitude (e.g. Pr(**e**) = f(*L*,*A*) for SCNA **e** of length *L* and amplitude *A*).

**Background Estimation:** Updates the background rate of SCNA formation (e.g. Pr(**e**) = f(*L,A*) ) given the sequence of SCNAs inferred from the current deconstruction.

**Details**

The deconstruction procedure amounts to choosing, from among an enumerated set of possible deconstructions, the one that is most probable given the estimate of the background rate of SCNAs. For simplicity, we assume that all SCNAs are independent, so the probability of observing a given set of SCNAs is equal to the product of the individual probabilities of observing each SCNA under the background model.


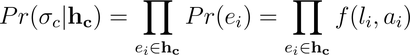


for SCNA events e_i_ of length l_i_ and amplitude a_i_.

At the start of the algorithm, the background rate of SCNAs (f(*L,A*)) is unspecified. We therefore begin by deriving an initial estimate of the background rates of copy number events across all samples using a highly simplified deconstruction procedure that leads to a unique set of SCNAs for each segmented chromosomal profile. This initial deconstruction procedure is based on two assumptions: 1) that each copy number breakpoint represents only a single copy number event, and 2) that copy number gains are never followed by copy number losses, and vice versa. Under these strict assumptions (which we relax later), the more extreme the amplitude of a copy number segment, the later it must have occurred during development. Thus, the evolutionary history of each chromosome can effectively be deconstructed “in reverse” by merging the most extreme amplification or deletion segment on each chromosome with its closest neighbor (recording the amplitude difference and segment length at each step) and repeating until the zero (or diploid) level has been reached (as shown in Supplemental Figure 1a).

At the end of each round of deconstruction, we bin ­the copy number events from all samples according to their length (expressed as a fraction of a chromosome arm, see below) and amplitude (expressed as change in copy number), and use the frequency of segments residing in each bin as an estimate of the background probability (f(*L,A*)) of such events in subsequent iterations. To avoid over-fitting the background probability distribution to the initial deconstruction, we smooth the distribution by adding uniformly distributed ‘pseudocounts’ to each bin (equal to 1% of the total number of SCNAs across the dataset).

For subsequent iterations, we expand the space of evolutionary histories that are considered by allowing for the existence of ‘basal’ copy levels about which the general Ziggurat Deconstruction procedure can be applied (see Supplemental Figure 1b). This expanded framework allows for deletion events to occur on top of the amplified ‘basal’ copy levels, and vice versa, generating more realistic deconstructions than the initial iteration. In principle, one could reconstruct all possible histories for a chromosome by fitting up to **n+1** ‘basal’ copy levels, where **n** is the number of segment breakpoints on that chromosome. An evolutionary model with **k ‘**basal’ copy levels can be specified by **2*k**-1 free parameters, representing the **k** copy levels and the **k-1** breakpoints between these levels. Because one can always obtain a better fit to the underlying data by increasing the number of free parameters, one must introduce a regularization term to compare models with different model complexities.

We introduce this regularization penalty using the Bayesian Information Criterion [45]. Specifically, for a proposed SCNA history **h_c_** with k basal levels, we calculate the BIC:


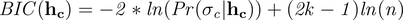


The proposed SCNA history with the minimal BIC is the model with the greatest explanatory power and the one that is chosen for each round of deconstruction.

In practice, we cap the number of ‘basal’ copy levels per chromosome to 2, both to limit the computational cost of the procedure and because the vast majority of cancer chromosomes appear to be well-fit by a maximum of 2 copy levels. Intuitively, these ‘basal’ levels can be thought to represent the copy levels of the two chromosome arms, although importantly, we do not constrain them to correspond as such by allowing the breakpoint between them to occur anywhere along the chromosome.

Because the underlying data are segmented, we simplify the search for the parameter values that maximize the likelihood function **Pr(σ_c_ | h_c_** ) by searching over values and breakpoints that are present in the data, and hence we need only consider a finite number of models for each chromosome representing all possible combinations of discrete parameter values. We therefore find the optimal model by enumeration over these finite possibilities. We first loop over the **n** possible model breakpoints (including the possibility that the chromosome has only a single level), and for each potential breakpoint find the ‘basal’ level or levels that maximizes the total likelihood of all segments given that breakpoint. We then choose the optimal breakpoint by finding the breakpoint whose best model has the minimal BIC.

In theory, one could continue to iterate between deconstruction and background estimation until the optimal parameter values do not change from one round to the next; although we have not formally proven that this procedure will converge on all datasets, we find that it does converge in practice. By default, we only perform two rounds of likelihood optimization, as we have found that the background distribution and deconstruction parameters tend to converge rapidly on most datasets; however, our code allows users to increase the number of iterations if desired.

To ensure that our procedure is not highly dependent on the initial deconstruction procedure, we tested the ZD procedure using 100 random initializations of the background rate and compared the resulting deconstructions. In every case, the output eventually converged to the same background distribution (data not shown), although in the majority of these cases several iterations were required. This suggests that our optimization procedure is not highly dependent on the initial deconstruction procedure used, and that the final deconstruction obtained by this procedure is likely to represent a robust optimum.

After the final deconstruction, we plot the distribution of segment lengths and amplitude differences across the entire dataset (see Supplementary Figure 2). To allow for the comparison of events occurring on chromosomes of different lengths, we normalize the length of each SCNA by calculating the fraction of each chromosome arm covered by the SCNA; for SCNAs that cross the centromere, the length is expressed as the sum of the fractions of each chromosome arm covered by the SCNA. As noted in the main text, the sharp increase in the frequency of segments occupying exactly 1 chromosome arms allows for the definition of a natural length-based definition of ‘focal’ and ‘arm-level’ SCNAs. Although the user can choose a different threshold, by default SCNAs covering greater than 98% of a chromosome arm are considered ‘arm-level’ while those covering less than 98% are considered ‘focal’.

1. **Probabilistic framework for scoring copy number events**

One of the novel aspects of the original GISTIC 1.0 algorithm [15] was that it weighted both the frequency and mean amplitude of copy number alteration at each locus when identifying significantly altered regions, rather than just the frequency of alteration. The original GISTIC G-score, defined as


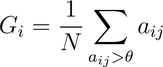


for marker *i,* sample log_2_ copy ratios *a_ij_,* and copy-ratio threshold θ, is equivalent to multiplying the frequency of alteration by the mean amplitude in altered samples. While this score captured the intuitive notion that higher amplitude changes are more likely to represent driver alterations than lower amplitude changes, it did not explicitly represent these likelihoods. Moreover, we wanted to extend the score to incorporate additional features of copy number events that may affect the background rates at which they occur, such as their length or chromosomal location.

We therefore set out to define a general framework for scoring the observed copy number changes in each region according to the negative log of the probability of observing such changes according to a specific background mutation rate of SCNAs. As described in the main text, this is a principled approach to defining a score in place of the arbitrary scores used in GISTIC 1.0 and other copy number methods, and allows for the modeling of variation in the background rate according to specific features of each SCNA.

**a. Scoring for individual markers**

The biological implication of the arm-level events is unclear, and they may very well target multiple genes or pathways [46]; therefore we are typically most interested in searching for regions that are significantly altered by focal events. However, the likelihood of observing a focal event may depend on whether a superimposed arm-level event is present. We therefore generate a score reflecting the probability of observing the set of focal SCNAs given the existence of the observed set of arm-level SCNAs. Let **B_i_** = {**b_1_, b_2_,** …} represent the set of arm-level SCNAs **b_i_** covering marker i, and **F_i_** = {**f_1_, f_2_,** …} represent the set of focal SCNAs **f_i_** covering marker i. We define the focal GISTIC score at marker i, FG_i_, as follows:


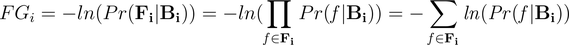
.

We assume in this formulation that the focal SCNAs are independent. One may define a similar score for arm-level SCNAs, although this raises additional issues not covered in the present manuscript. Interested readers are referred to [4].

Thus, all that remains is to approximate the function Pr(*f |* **B_i_**) for focal SCNA *f* of length *L* and amplitude *A* given a model for the background rate of SCNAs. One approach to this background mutation modeling is to approximate Pr(*f |* **B_i_**) by the frequency of occurrence of focal SCNAs of similar length, amplitude, and arm-level across a large dataset (similar to how the background rate is estimated during the ZD procedure, see above). However, as we described in the main text, this approach carries the potential to underweight driver events, which are generally of greater amplitude and shorter length than the typical SCNA (Supplementary Figure 3) and hence constitute the majority of events in their length/amplitude neighborhood. Thus, we set out to fit Pr(*f |* **B_i_**) to a functional form that would be relatively insensitive to the presence of driver events in the underlying dataset. To this end, we utilized a comprehensive dataset consisting of 3,131 cancer samples representing over 26 distinct histologic subtypes [4].

We started by noting that, across a wide range of cancers, the frequency of focal copy number events decreases inversely with the length for lengths up to a single chromosome arm (as in Figure 2b); this correlation is independent of amplitude for all but the highest amplitude copy segments (which are likely enriched for driver events). Since the likelihood that a marker is covered by a focal copy number alteration of any length less than a chromosome arm is roughly constant for all such lengths, the length of an SCNA contributes a constant factor to the log-likelihood of observing the data at a given marker under the background model. Moreover, except for the smallest length and highest amplitude bins, which are likely confounded by a relative abundance of driver events as well as platform-specific dynamic range, the frequency of a marker being covered by focal SCNAs reaching a given total amplitude in a sample decreases exponentially with amplitude [4]. In other words, we find that a reasonable null model for the probability that a marker *i* is covered by focal SCNAs of total amplitude A is given by:


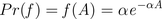


where α is a positive scaling parameter that is fit across all samples (and separately for amplifications and deletions).

The exponential relationship between observed frequency and event amplitude implies that the probabilistic score, defined as the negative log of the probability of observing the data, is linearly proportional to amplitude of the copy number change. As mentioned in the main text, one consequence of this change is an increased sensitivity to high copy-number changes that be highly sensitive to differences in platform dynamic range or variations in probe saturation kinetics as opposed to biologically important differences. To minimize the impact of these effects, we cap the copy number values prior to scoring the genome. Although the cap is technically a free parameter, in practice it can be reliably estimated from the data as the maximal copy value where the plot of log-frequency vs. copy number change is well fit by a linear curve. In our experience applying GISTIC 2.0 to sample sets run on multiple platforms, we have found that utilizing a cap greatly reduces the variability in inter-platform results.

Finally, we used our large cross-cancer dataset to model the dependence of focal copy number changes on the underlying arm-level changes by looking at the distribution of total focal copy number amplitudes as a function of total arm-level amplitude. We observed that focal amplifications were largely independent of their underlying arm-level changes; by contrast, focal deletions showed a strong dependence on arm-level amplitude, such that the probability of observing a focal deletion on top of arm-level change of amplitude –*B* was


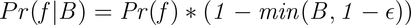


where we introduce ε (typically .05) for numerical stability in the exceedingly rare case that the arm-level copy change exceeds 1.

Thus, we derive a relatively simple model for the probability of observing focal copy number events at a given marker that depends only on the total amplitude of the copy change and underlying arm-level (for deletions):


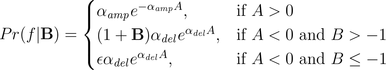


Using the probabilistic framework described above, we calculate separate focal GISTIC scores as follows:

1. Construct focal amplification and deletion genomes by summing the focal amplification and deletion SCNAs in each sample
2. Similarly construct arm-level amplification and deletion genomes by summing the arm-level amplification and deletion SCNAs in each sample
3. Score the observed amplification and deletion profiles in each sample according to –ln(Pr(*f |* **B))** as described above
4. Sum across independent samples to generate the final focal amplification and deletion scores

As with the original version of GISTIC, we calculate the distribution of focal GISTIC amplification and deletion scores expected by chance by random permutation of the marker locations throughout the genome, a procedure that also controls for variations in the rate of SCNA across different samples. One-sided p-values are calculated for each marker as the cumulative fraction of permuted score values that exceed that marker’s amplification or deletion score. Mutliple-hypothesis correction is performed using the Benjamini-Hochberg (BH) FDR method [36].

Finally, because the focal GISTIC score is represented a sum of independent random variables, we are able to utilize a semi-exact approximation method, as described in the original version of GISTIC [15], to derive an accurate approximation to the asymptotic distribution that would be achived by all possible permutations of marker positions. In this approach, the background distribution of focal GISTIC scores is derived by convolution of the distributions of focal GISTIC scores in each sample. As no actual permutations are performed, this allows us to calculate accurate p-values to levels of precisions that could not be computationally achieved by direct permutation.

**b. Scoring for genes**

As described in the main text, we also defined a modified scoring and permutation procedure (Gene GISTIC) that scores genes rather than markers. This procedure is designed to account for the likelihood of observing all deletion events affecting a single gene unit, even if those deletions are non-overlapping. The scoring procedure begins equivalently to the marker-based scoring defined above. However, for each sample, we first collapse the marker-based probabilities under the null into a gene-based probability as follows:


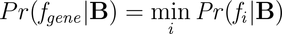


over all markers i covering a gene in each sample, and then generate the GeneGISTIC score for each gene by summing the logarithm of these gene-probabilities across independent samples.

Genes with greater numbers of markers are more likely to achieve a more extreme GeneGISTIC score by chance. We therefore modified our permutation procedure to account for the size of each gene when calculating the background distribution. Briefly, for a gene covered by *n*  markers, we first calculate the distribution of minimal marker scores in running windows of size *n* across each sample, and then convolve these sample distributions to derive a size-adjusted background distribution and p-value for each gene. As for marker-based scoring, these p-values are corrected for multiple hypothesis testing using the BH FDR method, albeit using a smaller number of hypotheses (the number of genes rather than the number of markers).

In the GeneGISTIC scoring framework, we define genes as those that encode proteins or miRNAs. Other classes of regulatory RNAs or non-genic functional units are not defined as genes and hence are not scored. In principle, one could include the entire genome under the GeneGISTIC framework by defining the markers within each inter-genic region as a single gene or as multiple independent genes. However, we prefer to not include such regions as it results in a less uniform interpretation of the GeneGISTIC scores. In a similar fashion, one could go beyond genes to define “metagenes” or pathways, although in this case simply taking the minimal copy value across all markers or genes in a pathway is unlikely to accurately reflect the activation state of an entire pathway; thus, more complicated scoring procedures may need to be devised. Moreover, including non-contiguous “genes” would require the computation of a much more complicated permutation procedure. Thus, while we believe this approach holds promise for incorporating additional biological knowledge into our procedure, significant work remains to be done.

1. **Identification of independently targeted regions of copy number alteration**

As described in the main text, GISTIC 1.0 utilizes a greedy segment peel-off algorithm to determine the number of independently significant SCNAs on each chromosome. Briefly, this process proceeds as follows:

1. Identify the most significant marker (or gene) on each chromosome (e.g. the marker (or gene) with the minimal FDR q-value < 0.25) and the segments that overlap it.
2. Subtract these segments from the data and rescore the chromosome.
3. Repeat from step 1 until no region crosses the significance threshold

This is a stringent procedure designed to ensure that regions that are closely linked to true target genes are not falsely discovered as significant. However, this greatly reduces the sensitivity of GISTIC to discover real secondary driver events the closer they get to another true target gene (see Figures 4a,b). Indeed, because SCNAs can target multiple nearby driver genes, we reasoned that a less greedy approach that reflects this biological reality might increase GISTIC’s sensitivity without significantly increasing its false discovery.

The approach we developed, termed ‘Arbitrated Peel-off’, bears a general similarity to the standard peel-off procedure described above. However, rather than assign segments to the first peak they cover, we allow for the segments to be split between multiple peaks, effectively reducing the amount that is subtracted from the score during step 2 of the peel-off procedure.

We formalize this process by defining an SCNA weight variable, **w_ij_**, which represents the amount of the score of SCNA **i** assigned to peak region **j**. Initially, **w_ij_** = 0 for all **i** and **j**. During the peel-off procedure, we assign and update **w_ij_** for each SCNA **i** covering the current peak region **k** through the following two procedures:

1. **Uncontested Assignment**

If **w_ij_** = 0 for all j, set **w_ik_** = **s_i_** for every segment **i** that covers peak **k**. In other words, if SCNA **i** has not previously been assigned to a peak, assign its entire score to the current peak **k**. Note that we assign all such uncontested SCNAs prior to moving on to step 2 (“Arbitrated Assignment”).

1. **Arbitrated Assignment**

Otherwise, let **C_i_ =**  be the set of all significant peak regions covered by a contested SCNA **i** together with the current peak **k** (SCNA **i** is called a ‘contested SCNA’, because multiple peaks in addition to the present peak can claim a fraction of the SCNA **i**’s weight) and let **C** be the union of all **C_i_**. We define the disjoint score **D** for each peak **c** in **C** as:


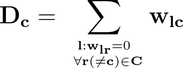


In other words, the disjoint score for a peak is the sum of the weights of all segments assigned to that peak, not including the contested segments. This gives a direct measure of the amount of evidence supporting peak region **c** independently of the contested SCNAs.

For each contested SCNA **i,** we then update **w_ij_** for all **j** in **C_i_** as follows:


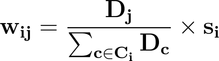


In other words, we split the entire weight of each contested SCNA among all the peaks it covers, each peak receiving a share of the SCNA in direct proportion to its disjoint score.

At the end of arbitrated assignment, we recalculate the score assigned to each peak region **j**:


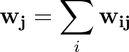


Thus, the arbitrated peel-off algorithm proceeds as follows:

1. Identify the most significant marker (or gene) on each chromosome (the marker (or gene) with minimal q-value, even if the q-value is > 0.25) and the set **S** of all SCNAs that overlap it.
2. Update the SCNA weight variable **w_ij_** for all SCNAs **i** in **S** using the procedure described above and recalculate the score assigned to each peak.
3. If the updated score of the current peak exceeds the significance threshold (e.g. updated FDR q-value < 0.25), record the peak as significant and repeat from step 1.
4. If not, do not record the current peak, and move on to the next chromosome.

Note that the additional sensitivity of arbitrated peel-off compared to the standard peel-off approach results from the way in which we allow previously peeled away SCNAs to contribute to a peak’s score (steps 1 and 2) before determining whether it is significant (steps 3), whereas previously the peel-off procedure would terminate as soon as an insignificant peak was identified. This potential increase in the score of newly identified peak regions can turn peaks that did not reach significance using the original, greedy peel-off method into significant peaks.

Conversely, one consequence of this procedure is that the score of previously peeled-off peaks decreases with the identification of adjacent peaks, as segments previously assigned uniquely to it are split among multiple peaks. One concern is the possibility that a previously significant peak could have enough weight taken away from it that it will no longer be significant. However, the use of the disjoint scoring metric ensures that a strict ordering of peaks is maintained throughout the arbitration process: peaks identified earlier in the peel-off process will always emerge with a marginally greater score than peaks obtained later. This guarantees that the arbitration process can never result in a situation where a previously significant peak becomes insignificant at the same time that an insignificant peak is made significant.

1. **Determination of boundaries of significantly altered regions**

The last step in the GISTIC 2.0 pipeline is the determination of the boundaries of each peak region that are likely to harbor the gene or gene(s) being targeted. As we discuss in the main text, previous copy number algorithms frequently used the MCR (minimal common region) as the region most likely to contain the target gene. The previous version of GISTIC used a related procedure, termed ‘leave-k-out’, that allows for at most **k** passenger events to be aberrantly defining the MCR. Indeed, the ‘leave-k-out’ boundary is defined by the widest left and right boundaries of the MCRs obtained after removing all possible combinations of **k** samples from the dataset (for more details, see [15]).

Here we describe “RegBounder”, a more principled approach to peak region boundary determination that is based on approximating the amount of expected local variation in GISTIC score profiles around a local G-score peak (in this framework, the G-score peak corresponds to the MCR). Unlike the MCR and ‘leave-k-out’ procedures, RegBounder uses information from all the SCNAs in a region, including the amplitudes of those SCNAs, to help define the boundaries for a peak region, yielding greater sensitivity and specificity (as described in the text).

First, let us define **G^k^** to be the GISTIC score profile generated by summing the segments assigned to peak region **k** during the peel-off procedure:


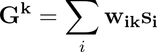


and let **G^k^_max_** represent the peak score in **G^k^** (analogous in this framework to the score at the MCR), let **i^max^_left_** and **i^max^_right_** represent the left and right boundaries of the peak score **G^k^_max_**, and let **G^k^_boundary_** represent the G-score corresponding to any current proposed boundary level.

We also define the passenger GISTIC score profile, **G^p^**, as the GISTIC score profile one would observe if all SCNAs were passengers. We approximate **G^p^** from the entire dataset by averaging the GISTIC score profiles generated by random permutation of the SCNAs across the genome in each sample. In contrast to the permutation procedure used to calculate the GISTIC p-values, here we are permuting entire segments and not just markers to capture the extent of local correlation expected in random GISTIC score profiles.

We next define the range distribution, **R(n)**, across the entire dataset as:


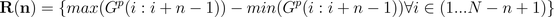


that is, the distribution of maximal score differences in **G^p^** in all running windows of size **n.** We are typically interested in a specific percentile of this distribution defined by the desired confidence level γ with which target genes are identified. Thus, we define:


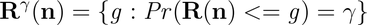


RegBounder works by iteratively narrowing the proposed boundary region until the actual difference between **G^k^_max_** and **G^k^_boundary_** is less than the γth-percentile of R(n) for the current boundary size n. These regions are predicted to contain the true target gene at least 100*γ percent of the time (see main text for validation).

Specifically, the RegBounder algorithm proceeds as follows:

For each peak region **k**:

1. Initially, let **G^k^_boundary_** = **G^k^_max_ – G_T_,** where **G_T_** represents the G-score required to an FDR q-value of 0.25. We can safely ignore SCNAs residing outside of the boundary region defined by this **G^k^_boundary_,** as the SCNAs within this boundary would have *been independently identified as a significant peak region without any of the neighboring SCNAs* and hence *must contain at least one true target gene.*
2. Define the initial left and right boundaries (LB, RB) as follows:


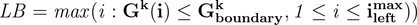


*
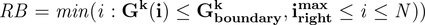
*

where N is the number of makers in the support of **G^k^** (e.g. where **G^k^** > 0).

1. Let n = RB-LB+1 be the number of markers in the current boundary, and calculate R^γ^(n) (see below).
2. If **G^k^_boundary_ > G^k^_max_ –** R^γ^(n), then stop. Otherwise, let **G^k^_boundary_ = G^k^_max_ –** R^γ^(n), and repeat from step 2. Note that because **G^k^_boundary_** is continually increasing, the regions will narrow until the termination condition is satisfied.

We note that because RegBounder utilizes simulation to compute the range distribution, it is theoretically stochastic, and hence the boundaries it produces may occasionally vary from run to run. However, with sufficient numbers of SCNAs and permutations, the error in determining R(n) becomes so small that the algorithm becomes for all practical purposes deterministic.

An additional issue is that for many datasets (especially those with high resolution markers), calculating R(n) for all window sizes n becomes computationally inefficient. We have therefore coded an alternative method for approximating R^γ^(n) which is based on pre-computing R^γ^(n) for n = 2,4,…,2^j^ where j = ceiling(log2(n_max_)) where n_max_  is the maximum number of markers on a given chromosome (representing the theoretically largest window size RegBounder would need to consider). We then fit R^γ^(n) for non-powers of two using spline interpolation. We have found that the error introduced in R^γ^(n) with this approximation is typically less than .1%, and for most datasets this error has no effect on RegBounder’s output or performance characteristics (data not shown) while dramatically speeding up the computation.

**RegBounder and GeneGISTIC**

Because adjacent genes in GeneGISTIC contain different numbers of markers, extending the RegBounder framework to GeneGISTIC is slightly more complex. In particular, there is not a one-to-one correspondence between the number of genes within a given boundary and the local variations in GeneGISTIC scores to be expected. In principle, one could estimate the range distribution for GeneGISTIC by redoing the size-controlled permutations on each of the SCNA permuted passenger G score profiles, although this would be computationally prohibitive for most datasets.

Thus, we have applied a simple transformation to the GeneGISTIC gene scores to allow RegBounder to be applied with greater simplicity. We do so by converting GeneGISTIC p-values into comparable marker-level scores by inverting the background distribution B for window-size n=1. In other words, we have the following situation:

B: score 🡪 p-value (B converts gene scores into p-values for a gene of size n)

B^-1^: p-value 🡪 score (B^-1^ converts p-values into gene scores, assuming n=1 for all genes)

Each marker covering a gene is given the same gene score (unless more than one gene covers that marker, in which case, the marker is given the maximal score of any gene they cover). In addition, we assign the markers between genes the maximal score of its neighboring genes. We then apply RegBounder to this marker-level score profile.

The final consideration is one of interpretation of the peak regions produced – for GeneGISTIC, boundaries can never occur in the middle of genes, whereas RegBounder performed on the marker space could produce boundaries in the middle of the gene. Thus, when using GeneGISTIC we require that a gene is contained entirely within a RegBounder peak region in order to consider it part of the peak; for regular GISTIC, we report any gene contained even partially within the RegBounder peak.

1. **Generation of simulated copy number datasets**

To validate the performance of “Arbitrated Peel-off” and “RegBounder”, we constructed a genome simulator that is capable of producing random datasets that closely mimic real copy number datasets (using some simplifying assumptions about how such data is generated). Our simulator allowed us to compare the performance of our algorithms as a function of several features, including sample size, driver frequency, and distance between two drivers on the same chromosome. Although we caution that our performance on simulated datasets does not guarantee similar performance on real datasets, these simulations are useful in comparing the effect of algorithmic modifications in a controlled environment in which the “ground truth” is known.

We base our simulated datasets on a reference set of 500 cancer samples randomly chosen from our large, cross-cancer dataset [4]. To construct a simulated genome of size n, we perform the following steps:

1. Randomly select n samples from the 500 reference dataset. We sample with replacement to allow for simulated datasets of greater than 500 samples.
2. For each sample, we first lay down n_i_ ‘passenger events’ for each chromosome, where n_i_ is the number of copy number events observed on chromosomes i=1,2,…,22, and X for the corresponding reference sample. For each chromosome, we select an equal number of passenger amplifications and deletions. The length and amplitude of each passenger SCNA is chosen at random from the collection of SCNAs in the reference sample, and the passenger is placed along the chromosome with uniform midpoint (for passenger SCNAs that would cross the telomere, we sacrifice uniformity slightly and slide the SCNA until its starting or ending point corresponds to one telomere).
3. We then lay down ‘driver events’ according to a frequency specified for each simulation. Primary driver events are always chosen to occur at the median position on the chromosome to minimize edge effects. For ‘arbitrated peel-off’ simulations, we also allow for the presence of a secondary driver with its own driver frequency.

The position of this secondary driver can be determined in one of two “modes”: in “length” mode, we fix the secondary driver to lie a fixed distance away from the primary driver. In “overlap” mode, we fix the percentage of overlap between primary and secondary driver, and choose the secondary driver position to be consistent with this specified overlap.

In either case, we first lay down primary driver events by choosing n = ceiling(frequency*sample size) samples to contain the driver. Because driver events are shorter and of greater amplitude than the typical passenger segment (see Supplementary Figure 3), we did not want to choose driver lengths/amplitudes from the reference data as we did for passengers. Rather, we select the driver length at random from an exponential distribution fit to the SCNA lengths surrounding known driver events in our reference dataset, and similarly, we select the driver amplitude from a log-normal distribution fit to the SCNA amplitudes surrounding known driver events (data not shown). We then position the driver event uniformly between (driver_position – driver_SCNA_length) and (driver_position+driver_SCNA_length) to ensure that each driver event covers the driver position.

We ran GISTIC2.0 on each of these datasets. For the arbitrated peel-off simulations, we ran GISTIC using either standard or arbitrated peel-off and RegBounder. For the RegBounder simulations, we ran GISTIC using arbitrated peel-off and either RegBounder, the MCR, or leave-k-out based boundary detection. Unless otherwise stated, the values shown for each data point in the results of our simulation represent 1,000 simulated datasets of the stated size.

The outcomes of these simulations were calculated as follows. For the arbitrated peel-off simulation, we define a driver gene as *“detected” by GISTIC* if GISTIC finds a boundary region containing the true driver locus. *Sensitivity* is defined as the average fraction of driver peaks recovered by GISTIC across all simulations with a given set of parameters. We define *independent recall of the secondary driver peak* to represent the fraction of times that GISTIC identified a peak at the secondary (less frequent) driver peak that did not also contain the primary (more frequent) driver peak. We define *dependent recall of the secondary driver peak* to represent the fraction of times that GISTIC identifies a peak containing both primary and secondary driver. *Total recall* is the sum of independent and dependent recall.

For the RegBounder simulations, we define sensitivity slightly differently. In this case, we care about the accuracy of the boundary detection algorithm GIVEN that a peak region near the driver locus was actually detected. Thus, we define a driver gene as *“detected” by GISTIC* if GISTIC finds a peak region within 5 Mb of the true driver locus. The *sensitivity* of a peak finding algorithm is thus the fraction of peaks for which the driver gene is contained within a boundary peak region divided by the fraction of detected peaks. *Peak size* is measured as the median number of markers contained within each boundary region.

To compare RegBounder to theoretical optimum, we computed for each simulation the distribution of distances between the driver peak and GISTIC MCR around “detected” peaks. We define the distribution of optimal, symmetric peak size by extending these MCRs in either direction until the driver gene has just been included in the boundary peak. We then compare the actual boundary peak sizes produced by RegBounder at a given confidence level γ to the γth percentile of the distribution of theoretically optimal symmetric peak sizes.

1. **Improved Memory Efficiency of Source Code**

Because GISTIC computes scores on a per-marker level, previous versions of the GISTIC source code expanded segmented copy number data into full m x n numeric arrays (where m is the number of markers on the measuring platform and n is the number of samples) to facilitate rapid computation of marker scores. However, we and others have found that this internal expansion resulted in very large memory requirements with the increasing resolution of array platforms (e.g. with increasing ‘m’) and with increasing large sample sets (e.g. with increasing ‘n’). We therefore set out to dramatically improve the underlying memory effiency of the GISTIC2.0 source code.

We accomplished this goal through the development of a MATLAB class, called a SegArray, which efficiently represents array-based data when one of the dimensions is segmented (and hence highly compressible). This encoding reduces the GISTIC2.0 memory requirements from O(m*n) [where m is the number of markers on the measuring platform and n is the number of samples] to O(s*n) [where s is the total number of segments identified]. Because the number of segments identified in a dataset is typically several orders of magnitude smaller than the number of markers (and, importantly, does not scale linearly with the marker number), this represents a major decrease in overall memory usage. For example, using the SegArray object we were able to reduce the physical memory requirements of running GISTIC2.0 on the TCGA GBM test set 85-fold (from 2.2 GB to 27 Mb) with only a modest increase in running time. The source code for the SegArray class is available for download along with the source code for the GISTIC2.0 package at [38].
