# Supplementary figures and images for: GISTIC2.0 facilitates sensitive and confident localization of the targets of focal somatic copy-number alteration in human cancers
Source: Genome Biol. 2011 Apr 28;12(4):R41. doi: 10.1186/gb-2011-12-4-r41 (PMC3218867; doi:10.1186/gb-2011-12-4-r41)

# Supplementary Figure 1

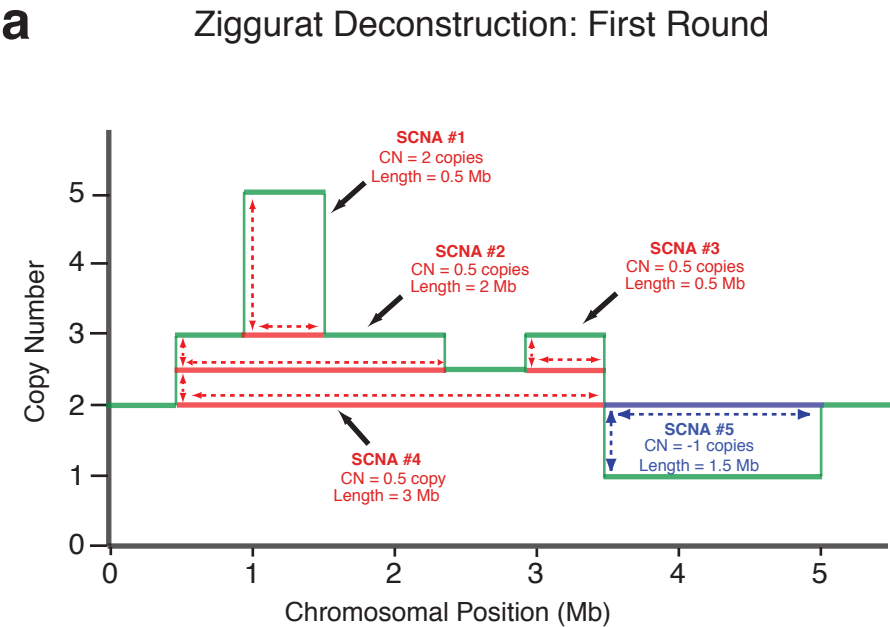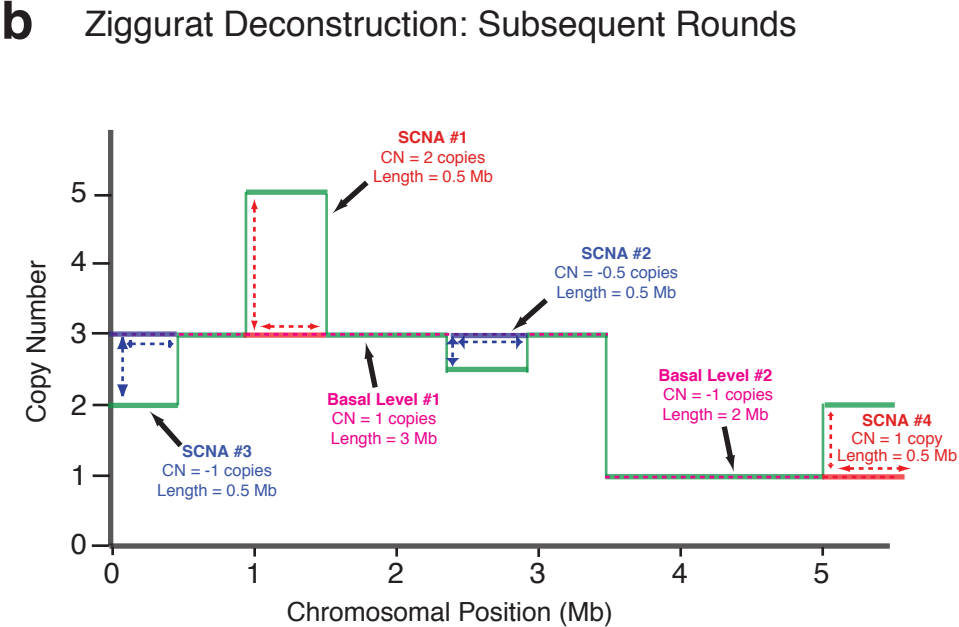

Supplement: Additional file 2 — Supplementary Figure S1: Ziggurat Deconstruction. (a) A hypothetical segmented chromosome (green line) is deconstructed with the simplified procedure used by Ziggurat Deconstruction (ZD) to initialize background SCNA rates. Dotted red and blue lines denote the length and amplitude of amplified and deleted SCNAs, respectively, while solid red and blue lines denote the result of merging the SCNA with the closest adjacent segment. (b) The same hypothetical segmented chromosome (green line) is deconstructed using the more flexible procedure of subsequent rounds of ZD. Here, the ZD is performed with respect to up to two basal levels (dotted magenta lines) that are fit to the data, allowing for amplified and deleted SCNAs to be superimposed. [file gb-2011-12-4-r41-S2.PDF]

## Supplementary Figure 2

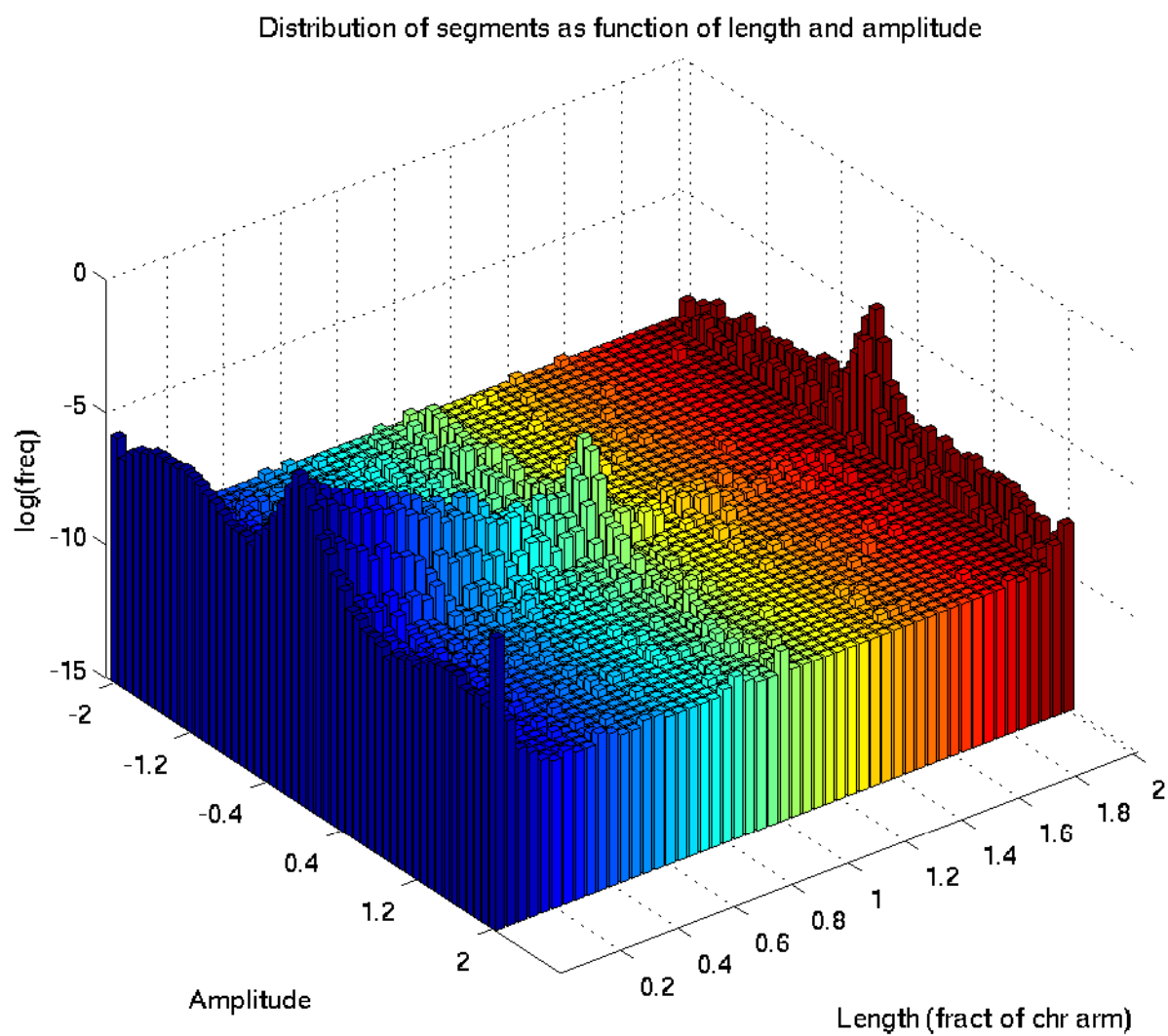

Supplement: Additional file 3 — Supplementary Figure S2: distribution of SCNA length and amplitudes. Two-dimensional histogram showing the frequency (z-axis) of copy number events as a function of length (x-axis) and amplitude (y-axis). Frequency is plotted on a log-scale to facilitate visualization of very low frequency copy number events. [file gb-2011-12-4-r41-S3.PDF]

Supplementary Figure 4

**a**

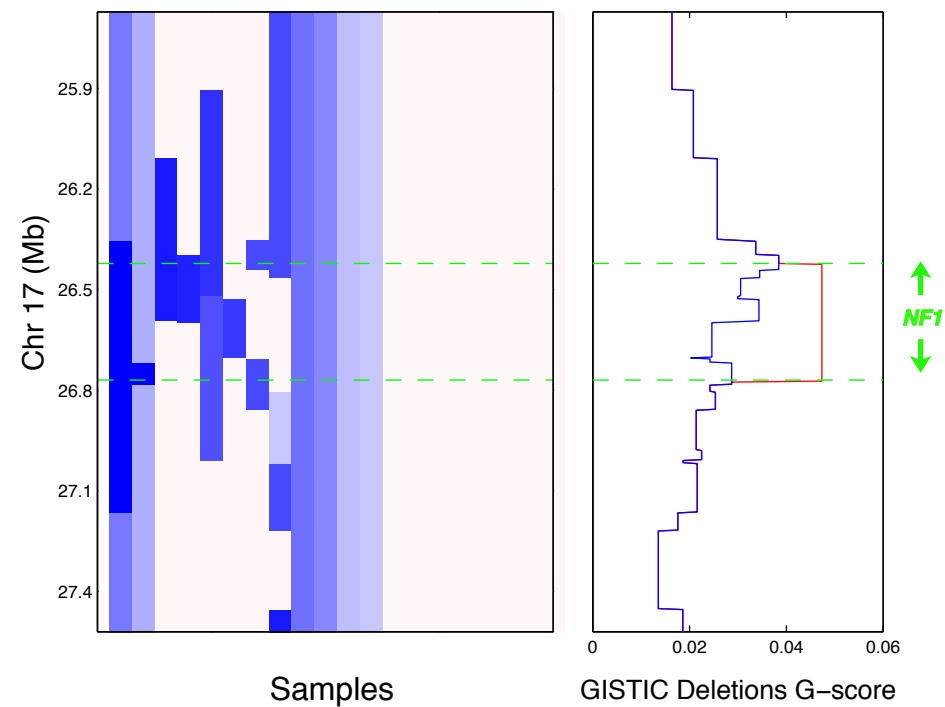

**b**

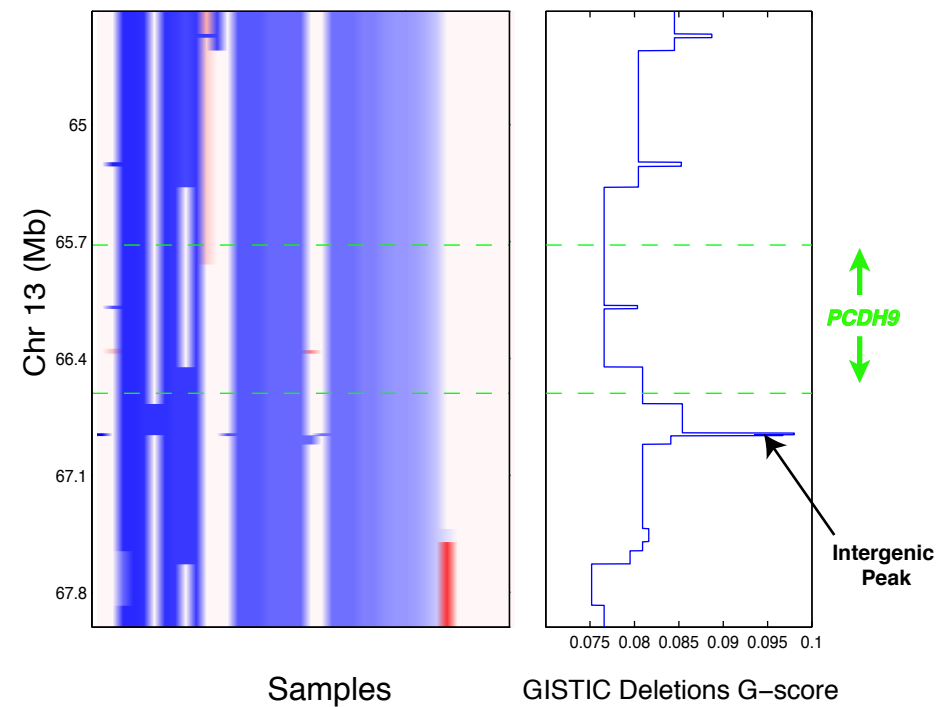

Supplement: Additional file 7 — Supplementary Figure S4: GeneGISTIC versus standard GISTIC. (a) GeneGISTIC helps identify genes subject to non-overlapping deletion, such as NF1. The left panel shows the 12 samples with focal deletions affecting NF1, many of which do not overlap. As a result, the standard GISTIC marker score (blue line, right panel) has multiple local maxima over NF1. By contrast, the GeneGISTIC score counts all of these deletions as contributing to the NF1 score, resulting in a score for NF1 (red line, right panel) that is significantly greater than that assigned to any of the individual markers covering NF1. (b) GeneGISTIC does not score deletions occurring outside of genes. The left panel shows a region of focal deletion occurring just outside the PCHD9 gene on chromosome 13. These deletions result in a peak in the markers deletion score (blue line, right panel) that is not detected by GeneGISTIC. [file gb-2011-12-4-r41-S7.PDF]
