# Supplementary material for: GISTIC2.0 facilitates sensitive and confident localization of the targets of focal somatic copy-number alteration in human cancers
Source: Genome Biol. 2011 Apr 28;12(4):R41. doi: 10.1186/gb-2011-12-4-r41 (PMC3218867; doi:10.1186/gb-2011-12-4-r41)

Supplementary Figure 3

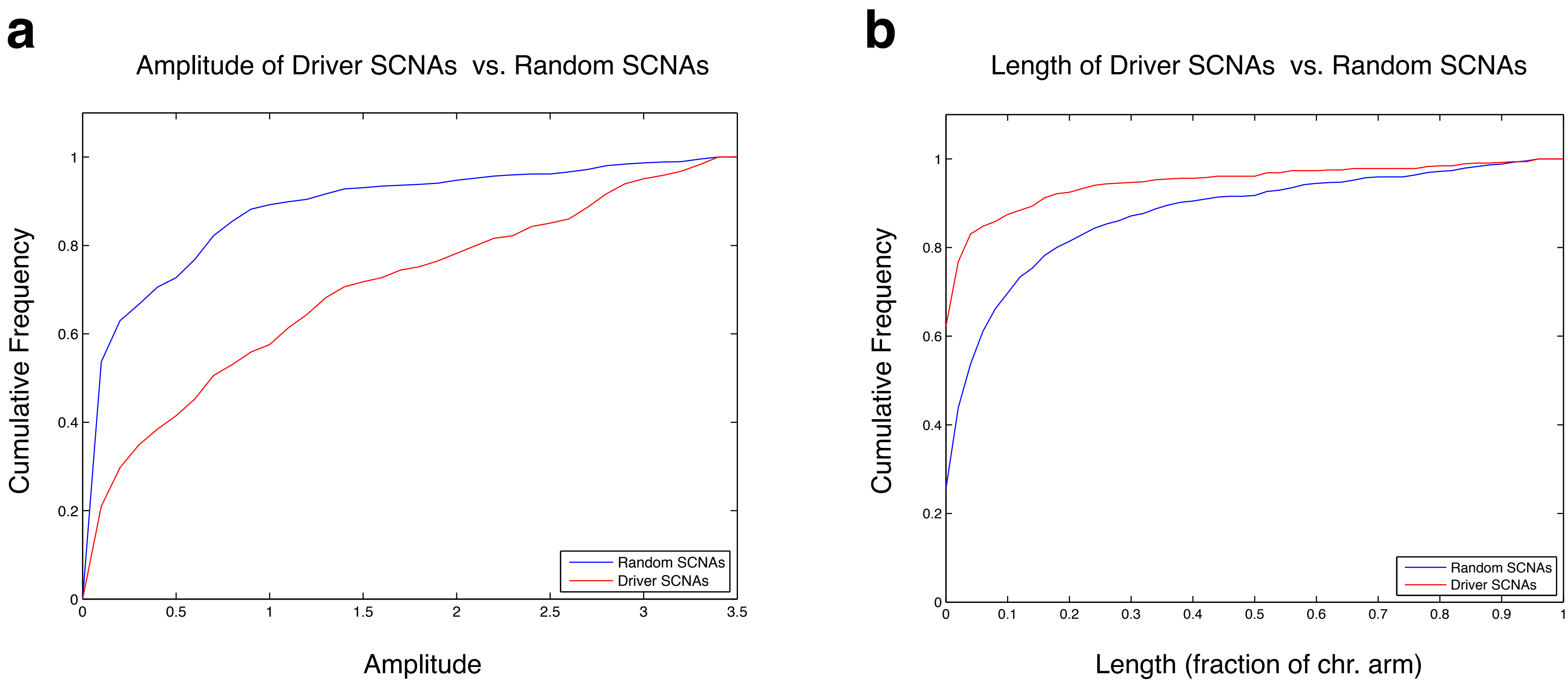

Supplement: Additional file 5 — Supplementary Figure S3: distribution of driver length and amplitudes. Driver SCNAs are typically of shorter length and higher amplitude than random passenger SCNAs. (a,b) Here we show the cumulative frequency distribution of SCNA amplitudes (a) and lengths (b) for SCNAs covering significantly amplified regions identified by GISTIC ('Driver SCNAs', red line) or by a similar number of randomly chosen non-driver regions ('Random SCNAs', blue line). [file gb-2011-12-4-r41-S5.PDF]
