# Supplementary material for: GISTIC2.0 facilitates sensitive and confident localization of the targets of focal somatic copy-number alteration in human cancers
Source: Genome Biol. 2011 Apr 28;12(4):R41. doi: 10.1186/gb-2011-12-4-r41 (PMC3218867; doi:10.1186/gb-2011-12-4-r41)

Supplementary Figure 5

Total Recovery of Secondary Driver Peak

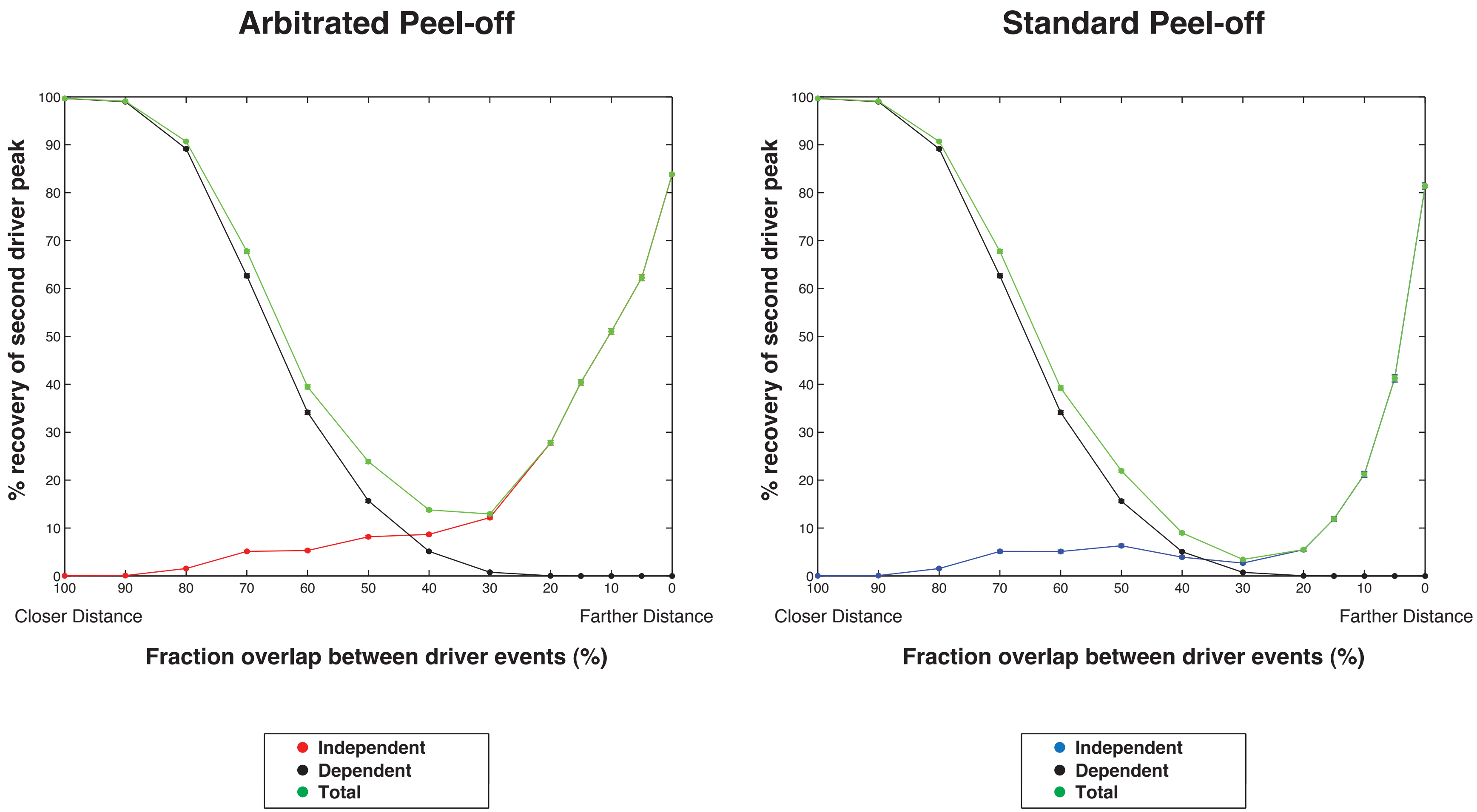

Supplement: Additional file 9 — Supplementary Figure S5: total recovery of secondary driver peaks. This figure shows the results from 10,000 simulations of 300 samples in which a primary driver event is present in 10% of samples and a secondary driver event is present in 5% of samples. In these simulations, we vary the fraction of overlap between driver events from 100% (total dependence) to 0% (total independence). Here we present to the total recovery of the secondary driver peak in GISTIC runs using arbitrated peel-off (left panel) or the standard peel-off (right panel). The red (left panel) or blue (right panel) lines show the fraction of secondary driver peaks identified in independent GISTIC peaks (that is, not containing the primary driver event), as is shown in Figure 4b. The black lines show the fraction of secondary driver peaks identified in dependent peaks (that is, a peak containing both the primary and secondary driver events), and the green lines show the total recall of secondary driver peaks (in any peak). Error-bars representing the mean ± standard error of the mean are drawn, but may be smaller than the point used to represent the mean and hence not be visible. [file gb-2011-12-4-r41-S9.PDF]
