# Supplementary material for: GISTIC2.0 facilitates sensitive and confident localization of the targets of focal somatic copy-number alteration in human cancers
Source: Genome Biol. 2011 Apr 28;12(4):R41. doi: 10.1186/gb-2011-12-4-r41 (PMC3218867; doi:10.1186/gb-2011-12-4-r41)

Supplementary Figure 6

RegBounder Peak Sizes vs. Theoretical Minimum Peak Sizes

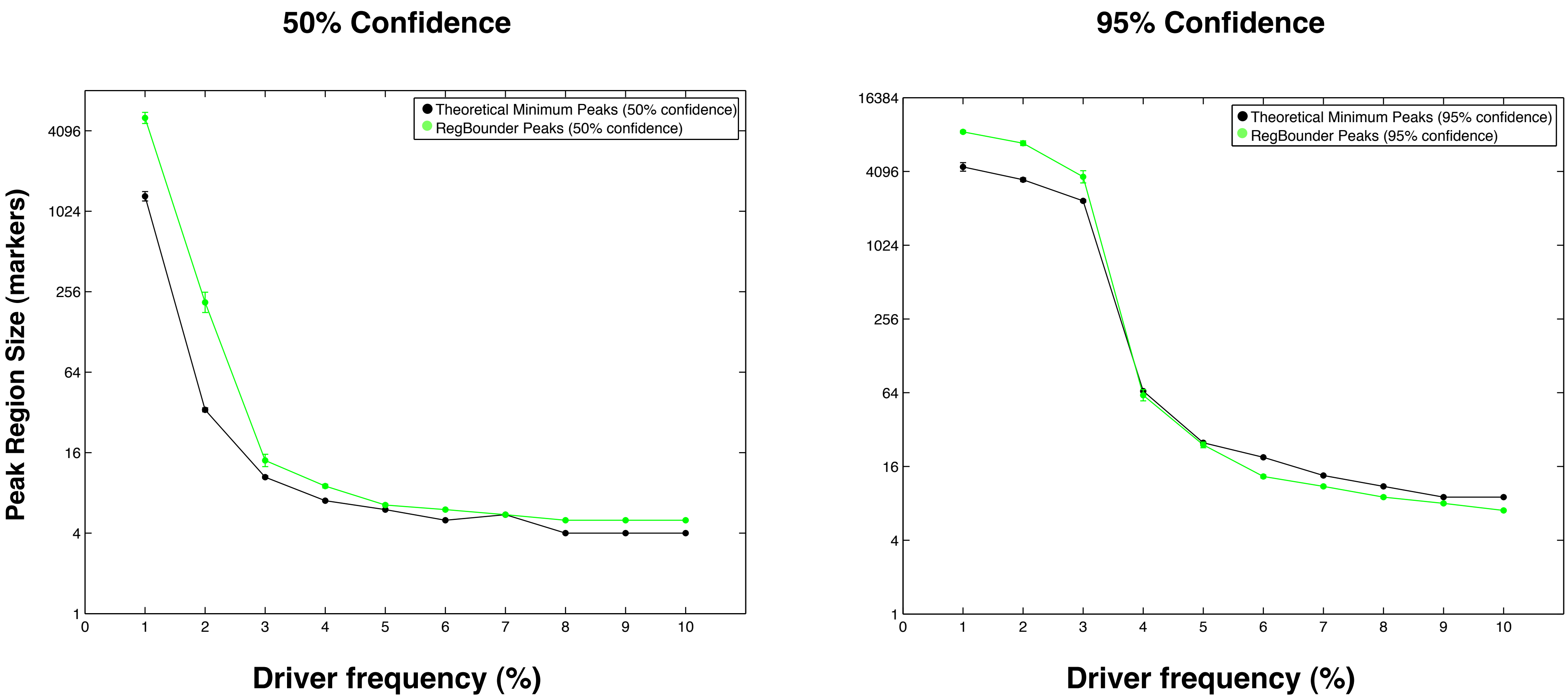

Supplement: Additional file 10 — Supplementary Figure S6: comparison of RegBounder to theoretically optimal peaks. Comparison between the peak region sizes obtained by RegBounder (green line) with the theoretically minimal peak region sizes (black line) that could be obtained by a similarly confident peak finding algorithm (Supplementary Methods in Additional file 1) at 50% (left) and 95% (right) confidence. Error-bars representing the median ± standard error of the mean are drawn, but may be smaller than the points used to represent the median and hence not be visible. [file gb-2011-12-4-r41-S10.PDF]
